# p–d orbital hybridization induced by transition metal atom sites for room-temperature sodium–sulfur batteries

**DOI:** 10.1093/nsr/nwaf241

**Published:** 2025-06-11

**Authors:** Hao Tian, Yaojie Lei, Bing Sun, Cheng-Chieh Yang, Chi-Liang Chen, Tao Huang, Xiaoyue Zhang, Yong Chen, Ailing Song, Le Pang, Hongxia Wang, Chung-Li Dong, Sean C Smith, Wei-Hong Lai, Yun-Xiao Wang, Xin Tan, Hao Liu, Guoxiu Wang

**Affiliations:** Centre for Clean Energy Technology, School of Mathematical and Physical Sciences, Faculty of Science, University of Technology Sydney, Broadway, NSW 2007; Centre for Clean Energy Technology, School of Mathematical and Physical Sciences, Faculty of Science, University of Technology Sydney, Broadway, NSW 2007; Centre for Clean Energy Technology, School of Mathematical and Physical Sciences, Faculty of Science, University of Technology Sydney, Broadway, NSW 2007; Department of Physics, Tamkang University, Tamsui 25137; Department of Physics, Tamkang University, Tamsui 25137; Centre for Clean Energy Technology, School of Mathematical and Physical Sciences, Faculty of Science, University of Technology Sydney, Broadway, NSW 2007; Institute for Carbon Neutralization Technology, College of Chemistry and Materials Engineering, Wenzhou University, Wenzhou 325035; Centre for Clean Energy Technology, School of Mathematical and Physical Sciences, Faculty of Science, University of Technology Sydney, Broadway, NSW 2007; Hebei Key Laboratory of Applied Chemistry, Hebei Key Laboratory of Heavy Metal Deep-Remediation in Water and Resource Reuse, School of Environmental and Chemical Engineering, Yanshan University, Qinhuangdao 066004; School of Chemistry and Physics, Faculty of Science, Queensland University of Technology (QUT), Brisbane, QLD 4001; School of Chemistry and Physics, Faculty of Science, Queensland University of Technology (QUT), Brisbane, QLD 4001; Department of Physics, Tamkang University, Tamsui 25137; Integrated Materials Design Laboratory, Department of Materials Physics, Research School of Physics, Australian National University, Canberra, ACT 2601; Laboratory of Advanced Materials, Shanghai Key Lab of Molecular Catalysis and Innovative Materials, Fudan University, Shanghai 200438; Institute of Energy Materials Science, University of Shanghai for Science and Technology, Shanghai 200093; Institute for Carbon Neutralization Technology, College of Chemistry and Materials Engineering, Wenzhou University, Wenzhou 325035; Integrated Materials Design Laboratory, Department of Materials Physics, Research School of Physics, Australian National University, Canberra, ACT 2601; Centre for Clean Energy Technology, School of Mathematical and Physical Sciences, Faculty of Science, University of Technology Sydney, Broadway, NSW 2007; Centre for Clean Energy Technology, School of Mathematical and Physical Sciences, Faculty of Science, University of Technology Sydney, Broadway, NSW 2007

**Keywords:** sodium–sulfur batteries, p–d orbital hybridization, single atom catalysts, electron transfer, sulfur conversion reaction

## Abstract

For energy storage applications involving sulfur redox reactions, uniformly dispersed metal sites in sulfur hosts serve as an effective approach to facilitate electron transfer during charge and discharge cycles. In this study, we exploited a facile method to construct transitional single-atom catalysts to overcome the kinetic limitations for electron transportation in room-temperature sodium–sulfur batteries. By the synergistic effect of polysulfide adsorption and p–d orbital hybridization between catalysts and intermediates, electron-donating and electron-capturing capabilities of different atomic sites towards sulfur redox reactions are systematically revealed. Remarkably, atomic Mn–N_4_ active moiety structures possess abundant unfilled antibonding orbitals, promoting p–d hybridization and leading to superior sulfur conversion reactions. This work establishes a design paradigm for single-atom catalysts in metal–sulfur batteries by linking atomic-scale electronic features to macroscopic performance. This atomic-level engineering strategy paves the way for high-energy-density room-temperature sodium–sulfur batteries, with potential extensions to other multivalent sulfur-based energy storage systems.

## Teaser

This work designs atomic-scale metal catalysts to boost sodium–sulfur batteries, improving efficiency, stability and battery lifespan.

## INTRODUCTION

The increasing demand for high-performance and sustainable energy storage systems has driven the exploration of new materials and chemistry to improve battery technologies [1,2]. Sodium-based batteries have gained significant attention owing to the abundance of sodium resources, cost-effective features and environmental friendliness, offering an attractive alternative to lithium (Li)–ion batteries [3,4]. In particular, room-temperature (RT) sodium–sulfur (Na–S) batteries become a significant option for energy storage solutions because of their high energy density (1274 Wh·kg^−1^) and potential to operate without the high temperatures required for traditional Na–S systems [5,6]. However, despite their advantages, RT Na–S batteries encounter some challenges, which hinder their commercial application, including the formation of highly soluble sodium polysulfides (Na_2_S_x_, 4≤ x ≤ 8), which leads to active material loss, interfacial degradation and rapid capacity decay due to their dissolution and reactions with electrolytes [7–11].

To address these challenges, the use of single-atom catalysts (SACs) for the application of RT Na–S batteries has attracted significant interest. SACs are characterized by isolated metal atoms dispersed on support materials, offering highly tunable catalytic properties and maximizing the utilization of active sites [12,13]. These catalysts are particularly promising for RT Na–S batteries because they provide an opportunity to fine-tune the electronic structures at the atomic scale, enabling efficient adsorption and conversion of sodium polysulfides, even at lower temperatures [14,15]. Monodispersed transition metal (TM) atoms can contribute to a pivotal role in enhancing the performance of the sulfur cathode in RT Na–S batteries. The introduction of TM atoms induces strong p–d orbital hybridization between the d orbitals of TM atoms and the p orbitals of sulfur species, facilitating efficient electron transfer and promoting polysulfide conversion [16–20]. This hybridization is crucial for enhancing the kinetics of polysulfide conversion, helping overcome the sluggish behavior typically observed in RT Na–S batteries. Additionally, TM-based SACs can effectively suppress the unwanted shuttle effect by enhancing the adsorption of intermediate polysulfides, preventing their diffusion to the anode, and improving the overall cycling stability of RT Na–S batteries [21]. While many significant studies have been undertaken so far, enhanced catalytic activities are still anticipated, emphasizing the necessity for rational regulation of the d-electron structure of metal centers through carefully chosen coordinated ligands and metal atoms [22–25]. Modifying electronic structures of catalysts significantly affects the energy barrier to reaction and binding abilities during the discharge and charge processes, highlighting the need to optimize the electrochemical properties for improved reversibility.

In this work, we systematically investigated multiple 3d TM SACs [manganese (Mn), iron (Fe), cobalt (Co), nickel (Ni), copper (Cu)] in RT Na–S batteries, providing a comprehensive comparison that varying d-electron configurations influence sulfur redox performance. A deeper mechanistic insight was provided through the dual-descriptor strategy, including the sulfur adsorption energy on the SACs and the p–d orbital hybridization strength, which enables design of high-performance SACs for Na–S batteries to achieve the best overall performance. In addition, advanced characterization techniques, specifically *in situ* synchrotron X-ray diffraction (XRD) and *ex situ* X-ray absorption spectroscopy (XAS) have been applied to simultaneously track the transformation of sulfur species and the electronic structure evolution of SACs during battery cycling, thus offering direct experimental evidence linking electronic states to catalytic activity. Consequently, the RT Na–S batteries assembled with sulfur@manganese–nitrogen–carbon (S@Mn–N–C) SACs exhibit outstanding rate capabilities and long durability compared to other TM–N–C SACs (including Fe, Co, Ni and Cu). By leveraging the atomic-level dispersion for single manganese atoms, this work elucidates that the optimal utilization of Mn–N–C SACs can improve the fixation of intermediate polysulfides and enhance the solid–solid conversion kinetics. Ultimately, this intensive p–d orbital hybridization in S@Mn–N–C cathodes improves the electrochemical performance of RT Na–S batteries, endowing them with an extended cycle life and enhanced stability.

## RESULTS AND DISCUSSION

### Theoretical prediction of MN_4_ electronic structures for RT Na–S batteries

**Figure 1.**
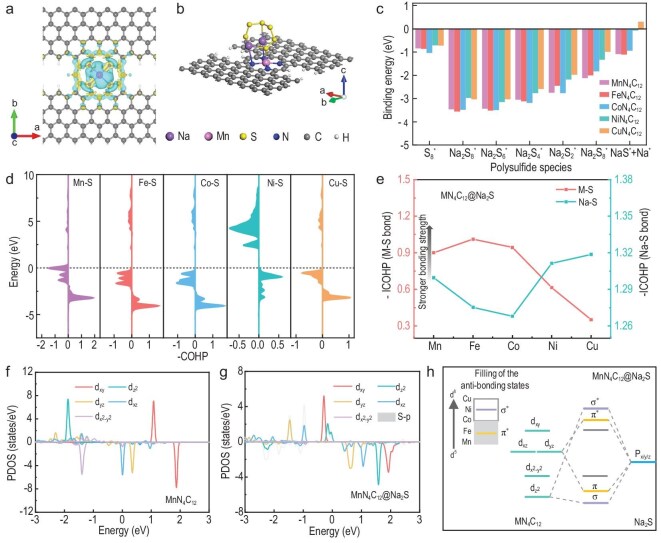
DFT calculations on polysulfide adsorption. (a) The calculated deformation charge densities of MnN_4_C_12_. The iso-surface level is 0.002 eV/Å^3^. The yellow and blue regions denote the positive area and negative areas, respectively. (b) Optimized molecular configurations of Na_2_S_6_ on MnN_4_C_12_. (c) The calculated binding energies of NaPS adsorbed on MN_4_C_12_. (d) The COHP for six MN_4_C_12_ candidates. (e) Strength of Na–S and M–S bonds elucidated by the ICOHP for the MN_4_C_12_@Na_2_S adsorption. Projected density of states (PDOS) of manganese d orbital of (f) MnN_4_C_12_ and (g) MnN_4_C_12_@Na_2_S. (h) Scenario of p–d orbital hybridization.

To understand the electrocatalytic polysulfide redox reactions on different MN_4_ (M = Mn, Fe, Co, Cu, Ni) surfaces, Density Functional Theory (DFT) calculations were conducted. Herein, we consider two MN_4_ simulation models with a pyrrole-type MN_4_C_12_ moiety (Fig. 1a) or a pyridine-type MN_4_C_10_ moiety (Fig. S1a) located in the interior of graphene sheets. Firstly, we studied the interaction between sodium polysulfides (NaPS) and MN_4_ surfaces. For all the NaPS species, the most stable adsorption configurations on MN_4_C_12_ and MN_4_C_10_ are similar. The sulfur prefers to bind to the M atom of MN_4_, and the sodium tends to bind to N atoms of MN_4_, as presented in Fig. 1b, Fig. S1b and Figs S2–S5 in the online supplementary file. Figure 1c and Fig. S1c show the calculated binding energies of various NaPS adsorbed on different MN_4_C_12_ and MN_4_C_10_, respectively. It can be clearly seen that both MN_4_C_12_ and MN_4_C_10_ moieties possess strong adsorption abilities for NaPS species. Additionally, the NaPS on MNC_12_ are generally binding more strongly than those on MNC_10_, and the binding strength of NaPS on MN_4_ follows the trend MnN_4_ ≈ FeN_4_ > CoN_4_ > CuN_4_ > NiN_4_. The calculation results demonstrate robust chemisorption of NaPS on MN_4_C_12_ and MN_4_C_10_ surfaces, which facilitate the NaPS reduction and restrict the shuttle effect. In addition, titanium and vanadium SACs are also predicted to exhibit even stronger polysulfide binding strength and faster conversion kinetics (Fig. S6), which agrees well with the reported literature [26], and molybdenum-based single-atom or cluster catalysts have shown promise in Li–S systems [27]. These would be exciting directions for future Na–S catalyst development.

In order to investigate the origin of the binding strength trend of NaPS on MN_4_, we introduced the projected crystal orbital Hamilton population (pCOHP) derived from the overlap-population-weighted densities of states to evaluate the bonding–antibonding behaviour of M–S bonds and calculated the integral of COHP (IpCOH) to quantify the M–S bond. As shown in Fig. 1d and Fig. S1d, the bonding orbital shifts gradually above Fermi level from manganese to copper, which is consistent with IpCOH values that gradually decrease towards copper (Fig. 1e and Fig. S1e), demonstrating that the M–S bond weakens from manganese to copper.

To deeper understand the reasons for enhanced NaPS binding on MnN_4_, the d orbitals of manganese of MnN_4_ were investigated. It is widely recognized that the interaction between the d orbitals of TMs and the p orbitals of sulfur species play a pivotal role in shaping the binding properties and catalytic activities of SACs. Specifically, the 3d orbitals of TMs are categorized into five groups: d_z_^2^, d_xy_, d_xz_, d_yz_ and d_x_^2^_-y_^2^ (Fig. 1f and Fig. S1f). Of particular interest, the d_z_^2^ orbital, in conjunction with the d_xz_ and d_yz_ orbitals, engages in interactions with the p orbitals of sulfur within polysulfides (Fig. 1g and Fig. S1g). This interaction results in the creation of bonding states, which include sigma (σ) and pi (π) bonds, as well as the corresponding antibonding states, sigma star (σ*) and pi star (π*). With the increasing number of 3d electrons from manganese to nickel, the π* and σ* states are gradually occupied, as shown in Fig. 1h and Fig. S1h. Therefore, we expect that lower-atomic-number TM elements, which possess fewer occupied antibonding states, will be more proficient in the p–d orbital hybridization and will exhibit a stronger affinity for sulfur binding.

**Figure 2.**
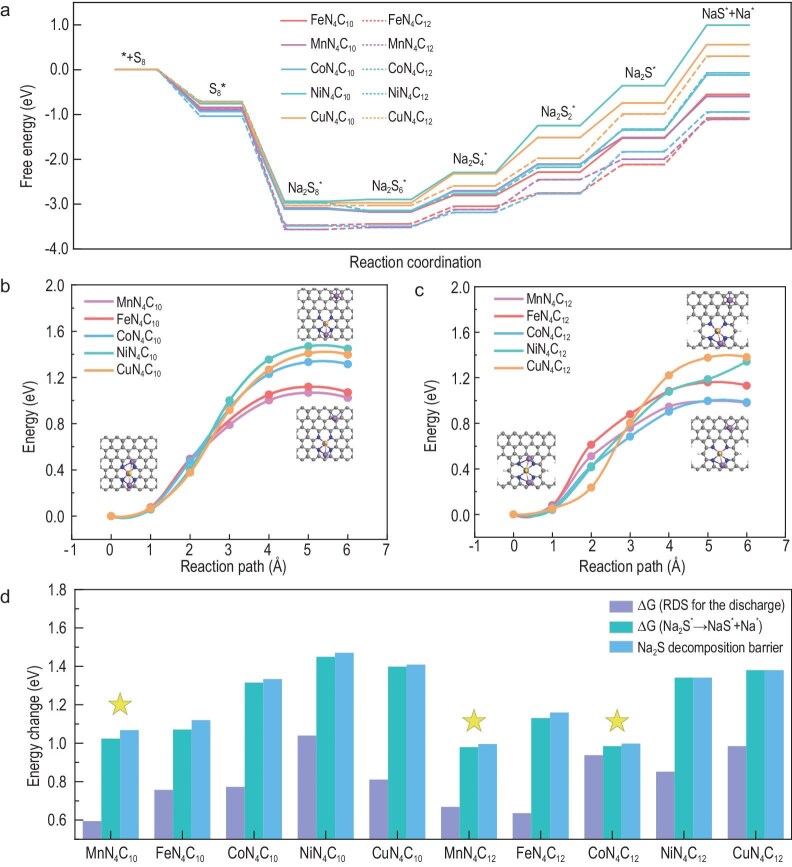
DFT calculations on polysulfide redox reactions. (a) The calculated Gibbs free energy profiles for the reduction of NaPS and the Na_2_S decomposition. The decomposition energy barriers of Na_2_S on (b) MN_4_C_10_ and (c) MN_4_C_12_. (d) Summary of the free energy changes of the RDS for the discharge process and the Na_2_S decomposition, and the decomposition barriers of Na_2_S on various MN_4_. The yellow stars indicate the three outstanding MN_4_ catalysts in Na–S batteries.

We then evaluated the electrocatalytic activity of polysulfide redox reactions on various MN_4_ surfaces. Since the reduction of polysulfides (reduction of S_8_ to Na_2_S) and the Na_2_S decomposition correspond to discharging and reverse charging processes, respectively, we calculated the Gibbs free energy profiles for NaPS reduction and the Na_2_S decomposition on various MN_4_ surfaces, as shown in Fig. 2a. The results show the formation of Na_2_S_2_ or Na_2_S is a rate-determining step (RDS) for the reduction of polysulfides, and the Na_2_S decomposition is endothermic on all the MN_4_ surfaces. Moreover, the reaction kinetics of the charging process on MN_4_ were also investigated by calculating the decomposition barriers of Na_2_S, as shown in Fig. 2b and c. The free energy changes of RDS for the discharge and the Na_2_S decomposition, and the decomposition barriers of Na_2_S on various MN_4_ are summarized in Fig. 2d. The DFT prediction of the electrocatalytic activities of polysulfide redox reactions on MN_4_ follow the trend CoN_4_C_12_ ≈ MnN_4_C_12_ ≈ MnN_4_C_10_ < FeN_4_C_10_ ≈ FeN_4_C_12_ < CoN_4_C_10_ ≈ NiN_4_C_12_ < CuN_4_C_10_ ≈ CuN_4_C_12_ < NiN_4_C_10_ and, more importantly, CoN_4_C_12_, MnN_4_C_12_ and MnN_4_C_10_ exhibited outstanding performance in Na–S batteries. Considering both MN_4_C_12_ and MN_4_C_10_ exist in our experimental samples, our simulation results are well consistent with the following experimental observations that the electrocatalytic activities of polysulfide redox reactions on MN_4_ catalysts follow the trend Mn–N–C > Co–N–C > Fe–N–C > Ni–N–C ≈ Cu–N–C.

According to the Sabatier principle and recent studies, optimal catalytic performance tends to be achieved at moderate binding energies, where the interaction strength is optimized—not too weak to limit adsorption and not too strong to hinder desorption [28]. MnN_4_ shows exceptional catalytic performance, which is attributed to its optimal polysulfide adsorption energy followed by efficient catalytic conversion. In contrast, the excessively strong binding energy of FeN_4_ impedes the release of intermediates, whereas NiN_4_ and CuN_4_ exhibit insufficient binding affinity, leading to poor polysulfide capture.

### Synthesis and characterization

The Mn–N–C catalyst was synthesized using a spatial pore confinement technique for creating SACs. Initially, bimetallic zeolite imidazolate frameworks (ZIFs) were produced following a previously reported method [29]. Room temperature mixing of zinc nitrate and manganese nitrate solutions with 2-methyl-imidazolate solution yielded ZnMn-ZIFs after 24 h of continuous agitation. This method can be adapted to create various bimetallic ZIFs, including ZnNi-ZIFs, ZnCo-ZIFs, ZnCu-ZIFs and ZnFe-ZIFs, which can be transformed into carbon materials at high calcination temperature in an inert atmosphere. These carbon particles are referred to as transitional SACs (M–N–C, where M represents Mn, Co, Ni, Cu or Fe).

**Figure 3.**
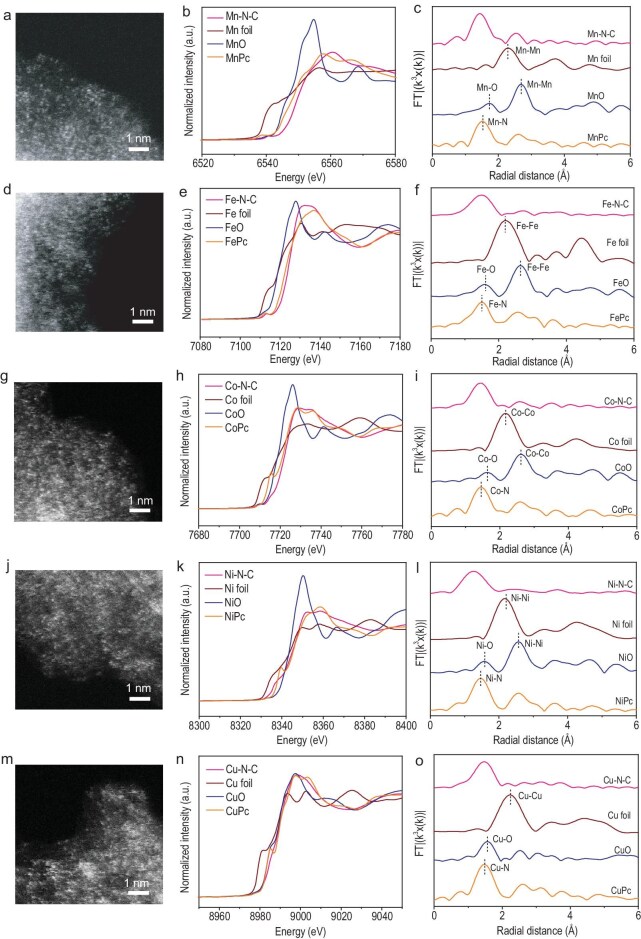
Synthesis and characterization of SACs. (a, d, g, j, m) High-resolution HAADF-STEM images of Mn–N–C (a), Fe–N–C (d), Co–N–C (g), Ni–N–C (j) and Cu–N–C (m). (b) XANES spectra and (c) FT EXAFS spectra in *R*-space of Mn–N–C, Mn foil, MnO and MnPc at the manganese K-edge. (e) XANES spectra and (f) FT EXAFS spectra in *R*-space of Fe–N–C, Fe foil, FeO and FePc at the iron K-edge. (h) XANES spectra and (i) FT EXAFS spectra in *R*-space of Co–N–C, Co foil, CoO and CoPc at the cobalt K-edge. (k) XANES spectra and (l) FT EXAFS spectra in *R*-space of Ni–N–C, Ni foil, NiO and NiPc at the nickel K-edge. (n) XANES spectra and (o) FT EXAFS spectra in *R*-space of Cu–N–C, Cu foil, CuO and CuPc at the copper K-edge.

Transmission electron microscopy (TEM) images at low magnification (Fig. S7a) and high magnification (Fig. S7b) revealed that the ZnMn-ZIF-derived carbon materials maintained their polyhedral structure after calcination, without visible metal aggregates, suggesting high dispersion of manganese atoms within the carbon structures. Energy-dispersive X-ray spectroscopy (EDS) images (Fig. S7c) showed homogeneous distribution of manganese, nitrogen, oxygen and carbon throughout the carbon framework, confirming that metal single atoms and nitrogen dopant are well-dispersed. The bright dots from aberration-corrected high-angle annular dark-field scanning transmission electron microscopy (HAADF-STEM) images (Fig. 3a) are displayed, indicating the presence of atomic dispersed manganese atoms. X-ray photoelectron spectroscopy (XPS) was employed to analyze the chemical environment of Mn–N–C SACs. Four peaks at 398.6, 399.6, 400.7 and 402.0 eV were deconvoluted in the high-resolution N 1s spectrum (Fig. S7d), which can be attributed to pyridinic-N, Mn-N, pyrrolic-N and graphitic-N [30]. Additionally, an ultralow manganese content of ∼0.6 wt% for Mn–N–C SACs was detected through inductively coupled plasma optical emission spectrometry (ICP-OES).

This synthetic approach can be expanded to produce other SACs, including cobalt, iron, copper and nickel. Polyhedral structures of the as-prepared SACs were well-preserved after calcination, which can be confirmed from TEM images of Fe–N–C (Fig. 3d and Fig. S8), Co–N–C (Fig. 3g and Fig. S9), Ni–N–C (Fig. 3j and Fig. S10) and Cu–N–C (Fig. 3m and Fig. S11). EDS mapping images demonstrated homogeneous distribution of carbon, metals (copper, cobalt, iron and nickel), nitrogen and oxygen within the carbon matrix. To examine the phase structure of M–N–C SACs, XRD measurements were conducted (Fig. S12). The XRD pattern revealed peaks at approximately 25.6° and 43.5°, which can be attributed to (002) and (100) planes of graphitic carbon. XPS results (Fig. S13) also presented the formation of metal–N bonds in M–N–C SACs. Additionally, M–N–C composites exhibited a combination of type I and IV isotherms from nitrogen adsorption–desorption (Fig. S14a) and comparable Brunauer–Emmett–Teller (BET) surface areas, as detailed in Table S1. Their size distribution (Fig. S14b) also revealed the existence of micropores.

The electronic structure and coordination environment of manganese sites were employed utilizing X-ray absorption near-edge structure (XANES) and extended X-ray absorption fine structure (EXAFS) measurements. The XANES spectra (Fig. 3b) demonstrated that the near-edge adsorption energy of manganese single atoms in Mn–N–C SACs, when compared to MnO, Mn_2_O_3_, MnPc and Mn foil, indicates a positive charge, consistent with previous XPS findings. The EXAFS spectra (Fig. 3c) exhibited a peak at ∼1.45 Å for both MnPc and Mn–N–C SACs, which can be attributed to Mn–N coordination [30,31]. The absence of a Mn–Mn peak around 2.31 Å in Mn–N–C SACs, compared to Mn foil, suggests atomic dispersion of manganese single atoms. EXAFS fitting curves in *R*-space and *k*-space (Fig. S15) indicated the coordination number of the Mn–N is determined to be 4 (Table S2,), suggesting a predominant MnN_4_ configuration in the carbon frameworks. The XANES profiles for Fe–N–C, Co–N–C, Ni–N–C and Cu–N–C SACs (Fig. 3e, h, k and n) revealed that the metals in M–N–C SACs are positively charged, with the oxidation states lying between those of the respective metal and metal oxides. The corresponding Fourier transform (FT)-EXAFS spectra of as-synthesized M–N–C SACs (Fig. 3f, i, l and o) showed no detectable metal–metal coordination, confirming the atomic dispersion of metals. EXAFS fitting results (Figs S16–S19) further validated the presence of the MN_4_ configuration in the synthesized SACs.

Sulfur encapsulation into SACs was conducted under an inert atmosphere to promote the sulfur infusion into the porous structures of SACs. The as-synthesized sulfur composites are denoted as S@M–N–C (where M represents Mn, Co, Ni, Cu, or Fe). TEM, HAADF-STEM and EDS images (Figs S20–S24) of the as-formed sulfur composite material (S@M–N–C) revealed that sulfur atoms were dispersed uniformly throughout the carbon frameworks. The corresponding aberration-corrected HAADF images also verified a homogeneous distribution of metal atoms after the sulfur encapsulation process. The sulfur content in S@Mn–N–C, S@Fe–N–C, S@Co–N–C, S@Ni–N–C and S@Cu–N–C was determined to be 53, 50, 43, 45 and 40 wt%, respectively, by thermogravimetric analysis (TGA) (Fig. S25).

### Electrochemical performance of S@M–N–C composites

**Figure 4.**
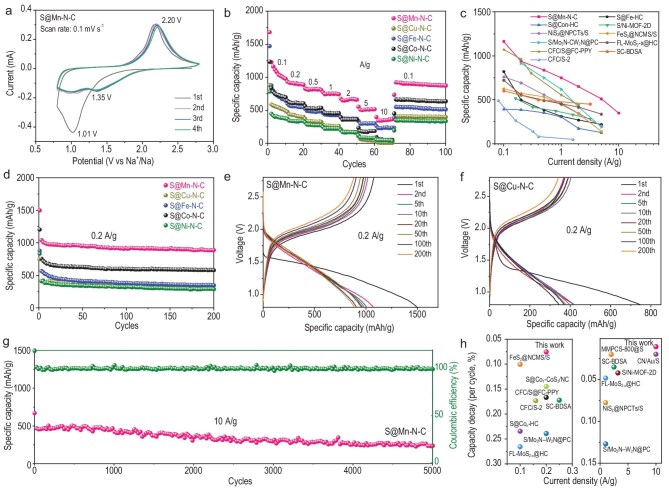
Electrochemical behaviors of S@M–N–C cathodes. (a) CV curves of the S@Mn–N–C electrode at a scan rate of 0.1 mV s^−1^ during the initial four cycles. (b) Rate capabilities of the S@Mn–N–C, S@Cu–N–C, S@Fe–N–C, S@Co–N–C and S@Ni–N–C electrodes. (c) Rate capability of S@Mn–N–C compared to other reported S-based electrodes for RT Na–S batteries. (d) Cycle performance of S@Mn–N–C, S@Cu–N–C, S@Fe–N–C, S@Co–N–C and S@Ni–N–C at a current density of 0.2 A g^−1^ for 200 cycles. Discharge–charge voltage profiles of (e) S@Mn–N–C and (f) S@Cu–N–C electrodes at a current density of 0.2 A g^−1^. (g) Long-term cycling ability of S@Mn–N–C at a high current density of 10 A g^−1^ through 5000 cycles. (h) Comparative cycling capability of S@Mn–N–C compared with previously reported sulfur-based cathodes for RT Na–S battery systems.

The electrochemical performance of the S@M–N–C composites was investigated as cathode materials for RT Na–S batteries. Cyclic voltammetry (CV) measurements of an S@M–N–C cathode (Fig. 4a and Fig. S26) displays a broad cathodic peak at 1.01 V in the first cycle, which could correspond to the generation of a solid electrolyte interphase (SEI) layer and sodium sulfide (Na_2_S) [32]. However, this peak vanishes in the subsequent discharge procedure, simultaneous with the presence of two reduction peaks at around 0.99 and 1.35 V, which derived from the electrochemical activation performance of the S@Mn–N–C electrodes during the sodiation process. In the subsequent anodic process, the peak at 2.18 V can be assigned to the reversible transformation from Na_2_S_2_ and Na_2_S to S [33]. Starting from the second cycle, the subsequent CV curves align very well, demonstrating the good electrochemical stability of the S@Mn–N–C electrodes. To better understand catalytic behavior of single manganese atoms to accelerate the transformation of sulfur to Na_2_S during the discharge process with the S@Mn–N–C electrodes, CV tests were also performed for the S@Co–N–C, S@Cu–N–C, S@Fe–N–C and S@Ni–N–C electrodes. Figures S26 and S27 illustrate the CV curves of S@Fe–N–C, S@Cu–N–C, S@Co–N–C and S@Ni–N–C presenting multiple redox peaks. However, only one broad peak was observed in the CV profile of the S@Mn–N–C electrode in the first cathodic and anodic scans. These results indicate that single manganese atoms can markedly catalyze the transformation reaction from S to Na_2_S and effectively promote the fast conversion of unstable NaPS. Figure S28 shows the discharge and charge profiles of the S@Mn–N–C electrode at different current densities. Both the discharge and charge processes exhibit stable voltage plateaus, aligning well with the characteristic peaks observed in the CV profiles. Figure 4b shows the rate performance of the various S@M–N–C cathodes. The S@Mn–N–C electrodes showed better rate performances compared to the rate capabilities of the S@Co–N–C, S@Cu–N–C, S@Fe–N–C and S@Ni–N–C electrodes. A high specific capacity of 1231 mAh g^−1^ can be achieved at 0.1 A g^−1^ by using S@Mn–N–C electrodes. When the discharge current reaches 10 A g^−1^, the battery can deliver a specific capacity of 361 mAh g^−1^. The manganese SACs can suppress side reactions of polysulfide intermediates via efficiently accelerating the consequent conversion processes towards short-chain products, which manifests in the superior kinetics (lower polarization in voltage profiles), high reversible capacity and excellent longevity of the Na–S cells. The superior rate performance of the S@Mn–N–C electrodes exceeds those of other reported sulfur-based cathodes for RT Na–S batteries, especially at high current densities (Fig. 4c and Table S3) [32–41].

The cycling performance of S@Mn–N–C electrodes at a current density of 0.2 A g^−1^ is shown in Fig. 4d. The S@Mn–N–C electrode exhibited the superior electrochemical behaviour after 200 discharge/charge cycles at 0.2 A g^−1^, achieving a high capacity of 888 mAh g^−1^ at 0.2 A g^−1^. A control cell with sulfur on nitrogen-doped carbon derived from ZIF-8 (Fig. S29) was also evaluated for RT Na–S batteries, and the S@N–C electrode can achieve a low specific capacity of 199 mAh g^−1^ at 0.2 A g^−1^ for 200 cycles, confirming that the MnN_4_ SAC is capable of catalyzing sulfur redox kinetics. In addition, a cell employing sulfur on nitrogen-doped carbon embedded with manganese nanoparticles (Mn NPs–N–C), derived from a ZnMn-ZIF precursor with a Zn:Mn molar ratio of 3:1 (Fig. S30a), was also evaluated in RT Na–S batteries. The S@Mn NPs–N–C electrode can only deliver a reversible capacity of 414 mAh g^−1^ at a current density of 1 A g^−1^ over 200 cycles (Fig. S30b). In contrast, the superior performance of the MnN_4_ SAC shows superior activity, highlighting its dual role in enhancing redox kinetics and effectively suppressing side reactions The discharge/charge profiles of the first, second, fifth, 10th, 20th, 50th, 100th and 200th cycles at 0.2 A g^−1^ of S@M–N–C cathode materials are shown in Fig. 4e and f and Fig. S31. The S@Mn–N–C cell (Fig. 4e) shows a long plateau at approximately 1.5 V during the initial discharge process, which is consistent with the characteristic peaks in the CV curves. Specifically, S@Mn–N–C electrodes exhibit a more pronounced high-voltage plateau, suggesting they rapidly initiate sulfur reduction, whereas S@Cu–N–C electrodes show a prolonged low-voltage plateau, consistent with slower kinetics in converting polysulfides to solid Na_2_S. Moreover, to further demonstrate the long cycle stability of the RT Na–S batteries, long-term cycling at a high current density of 10 A g^−1^ (Fig. 4g) was also investigated. The S@Mn–N–C electrodes showed an outstanding capacity of 232 mAh g^−1^ after 5000 cycles at 10 A g^−1^. Furthermore, Fig. S32 shows the cycling performance of the S@Mn–N–C cathode with a high areal loading of sulfur about 5 mg cm^−2^ at 1 A g^−1^ for 200 cycles, demonstrating stable cyclability. The SACs function within a well-engineered triple-phase environment (Fig. S33) comprising the SAC, liquid electrolyte and conductive carbon network, which are particularly conducive for RT Na–S batteries. This architecture ensures that even electronically insulating discharge products, such as Na_2_S or elemental sulfur, remain nanoscopic and well-connected to the conductive matrix, thereby preserving continuous catalytic activity. The remarkable cycling stability of the cell further indicates that the SAC active sites remain intact and unblocked over extended operation. Any Na_2_S or sulfur species that may temporarily adhere to the catalyst surface are readily reactivated and converted back to polysulfides or elemental sulfur during charging, confirming the reversible nature of the catalysis and the durability of the active sites. Figure 4h and Table S3 show comparisons of the long-term cycling stability obtained in this work and other reported results. Notably, the S@Mn–N–C cathodes demonstrated remarkable cycling performance among the candidate RT Na–S batteries.

Electrochemical impedance spectroscopy (EIS) spectra were conducted at the first discharge/charge cycle to uncover the charge-transfer kinetics of the S@M–N–C electrodes. The EIS curves of both S@Mn–N–C and S@Cu–N–C electrodes exhibited one semicircle in sequence with an inclined line in the discharging process. The deduced equivalent circuit is presented within the inset of Fig. S34. The resistance values obtained from Fig. S34 are summarized in Fig. S35. The resistance for the S@Mn–N–C electrodes first decreased and then became relatively stable for the remainder of the cycling processes, revealing the desolvation of sodium ions through the generation of the SEI layer [6] and the charge transfer process. Compared with the charge transfer resistance (R_2_) of the S@Mn–N–C electrodes, the S@Cu–N–C electrodes demonstrated lager resistance, revealing poor NaPS adsorption and sluggish kinetic transformation because of an elevated energy barrier and extended charge transfer pathway. In addition, the interface resistance (R_1_) of the S@Mn–N–C electrodes were lower than those of the S@Cu–N–C electrodes, indicating the higher electronic conductivity of the S@Mn–N–C electrodes.

**Figure 5.**
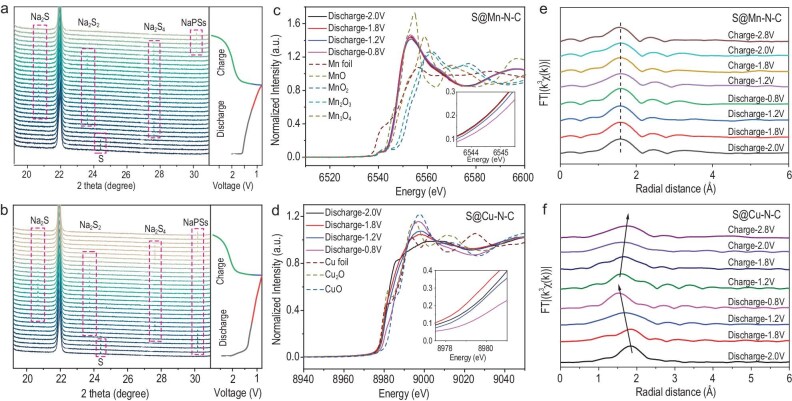
Understanding reaction mechanisms of S@M–N–C cathodes in RT Na–S batteries. (a) *In situ* synchrotron-based XRD patterns of an S@Mn–N–C electrode. (b) *In situ* synchrotron-based XRD patterns of an S@Cu–N–C electrode. (c) The manganese K-edge from *ex situ* XANES spectra of an S@Mn–N–C electrode during discharging. (d) The copper K-edge from *ex situ* XANES spectra of an S@Cu–N–C electrode during discharging. (The insets are details of the absorption energy shifts of the white-line peak). (e) *k*^3^-weighted FT-EXAFS curves of an S@Mn–N–C electrode in *R*-space during the discharge and charge processes. (f) *k*^3^-weighted FT-EXAFS curves of an S@Cu–N–C electrode in *R*-space during the discharge and charge processes.

To further explore the reaction mechanisms of S@Mn–N–C and S@Cu–N–C cathodes in RT Na–S batteries, the evolution of NaPS was investigated through *in situ* synchrotron powder XRD measurements (λ = 0.6884 Å) in the first discharge and charge processes (Fig. 5a and b). As shown in Fig. 5a, one weak peak at 20.6° can be observed in the XRD pattern before discharge, which can be indexed to the S element (PDF #: 00–064–0585). During the discharge process, diffraction peaks typically emerged at 23.6°, 24.4° and 27.6° indexable to Na_2_S (PDF #: 00-047-0178), Na_2_S_2_ (PDF #: 01-081-1771) and Na_2_S_4_ (PDF #: 00-027-0794). Notably, no long-chain NaPS were detected when S@Mn–N–C cathodes were used during discharge. In comparison, when S@Cu–N–C cathodes were used for RT Na–S batteries, long-chain Na_2_S*_x_* traces are initially observed, followed by the formation of Na_2_S_4_ and eventually Na_2_S_2_ and Na_2_S during discharging. These findings clearly show that manganese single atoms, via strong p–d orbital hybridization, can accelerate sulfur redox kinetics with efficient NaPS transformation during the discharge process, as well as an exceptional conversion capability of Na_2_S/Na_2_S_2_ during the charging process.

Furthermore, the *ex situ* XANES spectra of the S@Mn–N–C and S@Cu–N–C electrodes during the first cycle with the reference samples are illustrated in Fig. 5c and d, and Fig. S36a and b. In the manganese K-edge spectra for S@Mn–N–C electrodes (Fig. 5c and Fig. S36a), the peak at 6540.6 eV originated from the electronic quadrupole transition from 1s orbitals to vacant 3d orbitals as a result of p–d hybridization [14]. This indicates core-level electrons are excited to the valence shell. Conversely, no prominent peaks exist in the copper K-edge pre-edge spectra of the S@Cu–N–C electrodes (Fig. 5d and Fig. S36b).

As shown in Fig. 5c, the XANES spectra indicated that the oxidation state of Mn single atoms is +2 and the manganese K-edge shifts to a higher energy as S@Mn–N–C cells discharge from 2.0 to 0.8 V, indicating an increase in the valence states of manganese. This suggests strong p–d orbital hybridization, leading to a strong interaction between the manganese and sulfur atoms. During the discharge process, the hybridization process involves the five unpaired electrons from d orbitals of Mn²⁺ (3d^5^) overlapping with the p orbitals of sulfur. Mn^2+^ (3d^5^) has a half-filled d orbital, and the p–d hybridization results in partial electronic orbital overlap, forming bonding and antibonding states, which appear as π and σ bonds. The resulting π and σ bonds consist of contributions from both the d and p orbitals, showing strong covalent character. In the charging process, as the cell is charged to 2.0 V, the manganese K-edge shifts to a lower energy, indicating a reduction in the valence state of manganese. During this stage, the manganese atoms receive electrons from NaPS, accelerating their electron transportation (Fig. S36a). These observations align with the discharge and charge profiles, confirming that manganese atoms catalyze sulfur redox reactions during both the discharge and charge processes via electron transfer.

On the other hand, during the discharge process for S@Cu–N–C electrodes (Fig. 5d), the XANES spectra indicated that the oxidation state of copper single atoms is +2 and the copper K-edge shifts to lower energy from 2.0 to 1.8 V. Most of the electrons in the Cu^2+^ (3d^9^) d orbitals are paired, with only the d_yz_ orbital primarily involved in hybridization with the p orbitals of sulfur. The resulting π and σ bonds, along with their antibonding counterparts, are weaker for p–d hybridization in comparison to Mn^2+^ (3d^5^). During the charging (Fig. S36b), the copper K-edge initially shifts to higher energy and subsequently shifts back to a lower energy as the voltage rises from 2.0 to 2.8 V, which is less favorable for sulfur oxidation during charging.

Additionally, the EXAFS findings for S@Mn–N–C cathodes during discharge and charging (Fig. 5e) indicate that the average bond lengths of all Mn–N bonds in the first shell are approximately 1.57 Å, indicating that no phase changes are occurring during discharge–charge processes [42]. In contrast, for S@Cu–N–C cathodes (Fig. 5f), the bond lengths and the coordination value of Cu–N decrease during the discharging process, followed by the primary peak shifting back during the charging process. This highlights that S@Mn–N–C maintains a stable structure beneficial for preserving cycling stability, making it highly advantageous for long-term performance. The shifts in near-edge absorption energies highlight the different p–d hybridization abilities of various SACs, revealing their distinct catalytic properties.

The observed improvements in capacity, rate capability and cycling stability of Na–S batteries can be attributed to the stronger p*–*d orbital hybridization enabled by the TM SACs. These TM sites play a crucial role in anchoring and catalyzing the transformation of sulfur species in Na–S batteries. In contrast, host materials lacking accessible d orbitals fail to provide comparable interaction strength or catalytic functionality, leading to inferior electrochemical performance.

## CONCLUSION

In conclusion, we have demonstrated that p–d orbital hybridization induced by monodispersed manganese sites plays a crucial role in enhancing polysulfide conversion in RT Na–S batteries. By engineering the electronic structure of manganese-based SACs, the process of electron transfer can be greatly enhanced, significantly improving the adsorption and catalytic conversion of NaPS. This p–d orbital hybridization process plays a key role in accelerating solid–solid S conversion, which is the most fundamental challenge for RT Na–S batteries operated in carbonate-based electrolytes. Using advanced characterization techniques such as *in situ* synchrotron powder XRD and *ex situ* XAS, we confirmed that efficient catalyzation of manganese single atoms toward short-chain polysulfides enables high sulfur immobilization. This catalytic behavior is driven by the tailored electronic interactions of the manganese sites, which ensures uniform distribution and structural stability during sulfur conversion. The principles for Na–S catalysis including the requirement for moderate polysulfide binding and the beneficial functions of p–d orbital hybridization are expected to be applicable to other metal–sulfur batteries, such as Li–S batteries and emerging multivalent configurations (e.g. Mg–S, Zn–S batteries). Dual-atom catalysts such as Mn–Fe pairs may further improve polysulfide conversion by offering cooperative adsorption/catalytic sites, which will be explored in future studies. Our work demonstrates that Mn–N–C SACs are highly effective low-cost SACs, comparable to the reported Fe–N–C SACs. Moreover, our synthesis approach offers significant potential for large-scale production, further supporting its feasibility for practical applications. This study not only underscores the potential of SACs with unique electronic structures to surmount the challenges faced by RT Na–S batteries but also serves as an inspiration for future advancements in other energy-storage technologies. It thus paves the way for the rational design of next-generation, high-performance battery systems.

## Supplementary Material

nwaf241_Supplemental_File
