# Supplementary material for: p–d orbital hybridization induced by transition metal atom sites for room-temperature sodium–sulfur batteries
Source: Natl Sci Rev. 2025 Jun 11;12(7):nwaf241. doi: 10.1093/nsr/nwaf241 (PMC12247165; doi:10.1093/nsr/nwaf241)
Supplement: nwaf241_Supplemental_File [file nwaf241_supplemental_file.pdf]

Supporting Information

P-d orbital hybridization induced by transition metal atom sites for room-temperature sodium-sulfur batteries

Hao Tian†, Yaojie Lei†, Bing Sun†, Cheng-Chieh Yang, Chi-Liang Chen, Tao Huang, Xiaoyue Zhang, Yong Chen, Ailing Song, Le Pang, Hongxia Wang, Chung-Li Dong, Sean C Smith, Wei-Hong Lai, Yun-Xiao Wang*, Xin Tan*, Hao Liu, Guoxiu Wang*

Dr H. Tian, Dr Y. Lei, Dr B. Sun, T. Huang, Y. Chen, Prof. H. Liu, Prof. G. X. Wang
Centre for Clean Energy Technology, School of Mathematical and Physical Sciences, Faculty of Science, University of Technology Sydney, Broadway, NSW 2007, Australia.
Email: guoxiu.wang@uts.edu.au

C. Yang, C. Chen, Prof. C. Dong
Department of Physics, Tamkang University, Tamsui 25137, Taiwan

X. Zhang, Prof Xin Tan
Institute for Carbon Neutralization Technology, College of Chemistry and Materials Engineering, Wenzhou University, Wenzhou, Zhejiang 325035, P. R. China.
Email: xintan@wzu.edu.cn

X. Tan, S. C. Smith
Integrated Materials Design Laboratory, Department of Materials Physics, Research School of Physics, Australian National University, Canberra, ACT 2601, Australia

A. L. Song
Hebei Key Laboratory of Applied Chemistry, Hebei Key Laboratory of Heavy Metal Deep-Remediation in Water and Resource Reuse, School of Environmental and Chemical Engineering, Yanshan University, Qinhuangdao 066004, China

L. Pang, Prof H. X. Wang
School of Chemistry and Physics, Faculty of Science, Queensland University of Technology (QUT),
Brisbane, QLD 4001, Australia.

Dr W.-H. Lai
Laboratory of Advanced Materials, Shanghai Key Lab of Molecular Catalysis and Innovative Materials, Fudan University, Shanghai 200438, China

Prof Y.-X. Wang
Institute of Energy Materials Science
University of Shanghai for Science and Technology
Shanghai 200093, China
E-mail: yunxiaowang@usst.edu.cn

†These authors contributed equally to this work.

Experimental Section/Methods

Materials: The chemicals used in this work such as methanol (99%), zinc nitrate hexahydrate ($Zn(NO_3)_2 \cdot 6H_2O$), cobalt nitrate hexahydrate ($Co(NO_3)_2 \cdot 6H_2O$), nickel nitrate hexahydrate ($Ni(NO_3)_2 \cdot 6H_2O$), copper nitrate hemi(pentahydrate) ($Cu(NO_3)_2 \cdot 2.5H_2O$), manganese nitrate tetrahydrate ($Mn(NO_3)_2 \cdot 4H_2O$), iron sulfate heptahydrate ($FeSO_4 \cdot 7H_2O$) and 2-methylimidazole were purchased from Sigma-Aldrich and used as received without any further purification. Washing was achieved with ultrapure water and reagent grade ethanol and methanol where required. Ultrapure water was used for solution preparations.

Synthesis of ZIF-8. Typically, $Zn(NO_3)_2 \cdot 6H_2O$ (0.89 g) was dissolved in 30 mL of methanol to form a solution. 20 mL of methanol containing 2-methylimidazole (1.97 g) was poured into $Zn(NO_3)_2$ solution. The mixture was kept stirring at room temperature for 24 h. The resulting white precipitates was collected by centrifugation, washed with methanol three times, and finally dried at 80 ℃ overnight.

Synthesis of ZnMn-ZIF. Typically, $Zn(NO_3)_2 \cdot 6H_2O$ (0.743 g) and $Mn(NO_3)_2 \cdot 4H_2O$ (0.125 g) were dissolved in 30 mL of methanol to form a solution. 20 mL of methanol containing 2-methylimidazole (1.97 g) was poured into $Zn(NO_3)_2$ and $Mn(NO_3)_2$ solution. The mixture was kept stirring at room temperature for 24 h. The resulting white precipitates was collected by centrifugation, washed with methanol three times, and finally dried at 80 ℃ overnight.

Synthesis of ZnFe-ZIF. Typically, $Zn(NO_3)_2 \cdot 6H_2O$ (0.575 g) and $FeSO_4 \cdot 7H_2O$ (0.019 g) were dissolved into 50 mL of methanol to form a solution. 50 mL of methanol containing 2-methylimidazole (0.66 g) were poured into $Zn(NO_3)_2$ and $FeSO_4$ solution. The mixture was kept stirring at room temperature for 24 h. The resulting white precipitates were collected by centrifugation, washed with methanol three times, and finally dried at 80 ℃ overnight.

Synthesis of ZnCo-ZIF. Typically, $Zn(NO_3)_2 \cdot 6H_2O$ (0.863 g) and $Co(NO_3)_2 \cdot 6H_2O$ (0.0291 g) were dissolved in 30 mL of methanol to form a solution. 20 mL of methanol containing 2-methylimidazole (1.97 g) was poured into $Zn(NO_3)_2$ and $Co(NO_3)_2$ solution. The mixture was kept stirring at room temperature for 24 h. The resulting white precipitates were collected by centrifugation, washed with methanol three times, and finally dried at 80 ℃ overnight.

Synthesis of ZnNi-ZIF. Typically, $Zn(NO_3)_2 \cdot 6H_2O$ (0.743 g) and $Ni(NO_3)_2 \cdot 6H_2O$ (0.145 g) were dissolved in 30 mL of methanol to form a solution. 20 mL of methanol containing 2-methylimidazole (1.97 g) was poured into $Zn(NO_3)_2$ and $Ni(NO_3)_2$ solution. The mixture was kept stirring at room temperature for 24 h. The resulting white precipitates were collected by centrifugation, washed with methanol three times, and finally dried at 80 ℃ overnight.

Synthesis of ZnCu-ZIF. Typically, $Zn(NO_3)_2 \cdot 6H_2O$ (0.743 g) and $Cu(NO_3)_2 \cdot 2.5H_2O$ (0.116 g) were dissolved in 30 mL of methanol to form a solution. 20 mL of methanol containing 2-methylimidazole (1.97 g) was poured into $Zn(NO_3)_2$ and $Cu(NO_3)_2$ solution. The mixture was

kept stirring at room temperature for 24 h. The resulting white precipitates were collected by centrifugation, washed with methanol three times, and finally dried at 80 ℃ overnight.

Synthesis of single atom catalysts (metal-N-C SACs, metal: Fe, Co, Ni, Cu and Mn). The as-prepared ZIF nanoparticles were carbonized in flowing Ar in a tube furnace using a heating rate of 2 ℃ min^{-1} up to 800 ℃, dwell for 3 h.

Preparation of S@metal-N-C: The as-obtained black powder, metal-N-C, was mixed with S powder in a weight ratio of 1:1.5 and then sealed in a quartz ampoule. The sample in the final sealed quartz tube was annealed for 155 ℃ for 12 hours, and the temperature was then increased to 300 ℃ for 2 hours for a melt and diffusion approach.

Synthesis of S@Mn-N-C, S@Fe-N-C, S@Co-N-C, S@Ni-N-C and S@Cu-N-C. The Mn-N-C materials were ground in an agate mortar with S powder with a mass ratio of 1:1.5. Then, the mixture was sealed in an ampoule, inserted into a quartz tube, and heated at 155 ℃ for 12 h. The temperature was then increased to 300 ℃ for 2 h at the heating rate of 5 ℃ min^{-1}. The obtained sample was denoted as S@Mn1-PNC. Other S loaded single atom samples were obtained via the same procedures, the products of which were denoted as S@Mn-N-C, S@Fe-N-C, S@Co-N-C, S@Ni-N-C and S@Cu-N-C, respectively.

We focused on 3d transition metals ranging from Mn to Cu, as they are capable of forming stable MN_4 coordination sites within carbon matrices and collectively span a broad spectrum of d-electron configurations. This allows investigation of the d-band effect on polysulfide interactions. Early transition metals (e.g. Ti, V) and 4d metals (e.g. Mo) were excluded due to synthetic limitations and to ensure a consistent basis for comparison.

Electrochemical performance measurements. The electrochemical tests were performed via assembling half-cells in an argon filled glove box. 80% active materials (S@metal-N-C), 10% carbon black, and 10% carboxymethyl cellulose (CMC) were mixed with an appropriate amount of deionized water, and the slurry was coated on Cu foil and then dried in a vacuum oven at 60 ℃ overnight. In the following step, the electrodes were punched to form round disks with diameters of 0.97 cm, and the average mass loading was up to 1.6 mg/cm^{-2}. Sodium foil was used for both the reference and the counter electrode, while glass fibre was chosen for the separator. The electrolyte used for the Na-S batteries consisted of 1 M $NaClO_4$ in ethylene carbonate (EC)/propylene carbonate (PC) with a 1:1 volume ratio and 5% fluoroethylene carbonate (FEC) as additive. The sulfur cathode has a geometric area of about 0.78 cm^2 (10 mm in diameter). The average mass loading of cathode materials is up to 1.6 mg cm^{-2}. In order to fully exert the performance of S cathode, we used a sodium metal foil disc as the anode without limiting negative/positive (N/P) capacity ratio. The Na disc is about 1.13 cm^2 (~0.3 mm in thickness and 12 mm in diameter), showing a negative/positive (N/P) capacity ratio of roughly 18.3. The electrolyte-to-sulfur (E/S) ratio is about 50 μL mg^{-1} (that is ~25 μL of electrolyte for a 0.48 mg sulfur cathode).The

electrochemical measurements were performed on a NEWARE coin cell tester and a Biologic VMP-3 electrochemical workstation within the voltage range of 0.8–2.8 V with all capacities normalized by the weight of S.

Characterizations: The morphology and chemical composition of the as-prepared samples were observed by field emission scanning electron microscopy (FESEM, Zeiss Supra 55VP) and TEM (FEI Themis-Z Double-corrected 60–300 kV S/TEM). X-ray diffraction (XRD) measurements were carried out by using a scanning step of 0.04° per second in the 2θ range from 10° to 80° (Bruker D8 Discovery XRD). X-ray photoelectron spectroscopy (XPS) measurements were performed on an ESCALAB250Xi (Thermo Scientific, UK) equipped with mono-chromated Al K alpha (energy 1486.68 eV). The BET specific surface area and single-point pore volume were obtained from nitrogen adsorption isotherms measured at −196 ℃ using a nitrogen sorption instrument (ASAP 2460 Micropore Physisorption Analyzer). Prior to nitrogen adsorption measurements, the samples were degassed at 250 ℃ overnight. Raman spectra were obtained from a Renishaw inVia Raman spectrometer system (Gloucestershire, UK) equipped with a Leica DMLB microscope (Wetzlar, Germany) and a Renishaw He-Ne laser source producing 17 mW at 633 nm. X-ray absorption fine structure (XAFS) measurements of Mn, Cu, Fe, Co, and Ni K-edge were performed at the 1W1B beamline of the National Synchrotron Radiation Research Center in transmission mode. The X-ray was monochromatized using a double-crystal Si (111) monochromator and the energy was calibrated by metal foils. The XAFS data was analysed using the WinXAS3.1 program. Metal content in catalysts was determined by inductively coupled plasma optical emission spectrometry (ICP-OES).

DFT calculations. All of the spin-polarized density functional theory (DFT) calculations were performed using the VASP program,[1-3] which uses a plane-wave basis set and a projector augmented wave method (PAW) for the treatment of core electrons.[2] The Perdew, Burke, and Ernzerhof exchange-correlation functional within a generalized gradient approximation (GGA-PBE)[4] was used in our calculations, and the van der Waals (vdW) correction proposed by Grimme (DFT-D3)[5] was employed due to its good description of long-range vdW interactions. For the expansion of wavefunctions over the plane-wave basis set, a converged cutoff was set to 500 eV. In order to simulate different MN_4 (M=Mn, Fe, Co, Ni, Cu) moieties located in the interior of graphene sheets, we considered two MN_4 simulation models with pyridine-type MN_4C_{10} moiety (**Figure 1a**) or pyrrole-type MN_4C_{12} moiety (**Figure 1b**). The vacuum space was set to larger than 20 Å in the z-direction to avoid interactions between periodic images. In geometry optimizations, all the structures were relaxed up to the residual atomic forces smaller than 0.02 eV/Å, and the total energy was converged to 10^{-5} eV. The Brillouin zone integration was performed on the (2×2×1) Monkhorst–Pack k-point mesh.[6]

The Gibbs free energy ΔG of each reaction step in electrocatalytic sulfur reduction reaction (SRR) and Na_2S decomposition was calculated by:

$$\Delta G = \Delta E + \Delta ZPE - T\Delta S \quad (1)$$

where ΔE is the binding energy calculated by DFT, which is defined as $\Delta E = E_{ads/surf} - E_{ads} - E_{surf}$. Here, $E_{ads/surf}$, E_{ads} and E_{surf} are the total energies of adsorbate on the surface, adsorbate and clean surface, respectively. ΔZPE is the zero-point energy correction and $T\Delta S$ is the entropy correction, which were given in **Table S4**. The energy barriers for Na_2S decomposition on MN4 were calculated with the climbing-image nudged elastic band (CI-NEB) method,[7] in which all of the force components perpendicular to the tangent of the reaction path were relaxed to be less than 0.05 eV/Å.

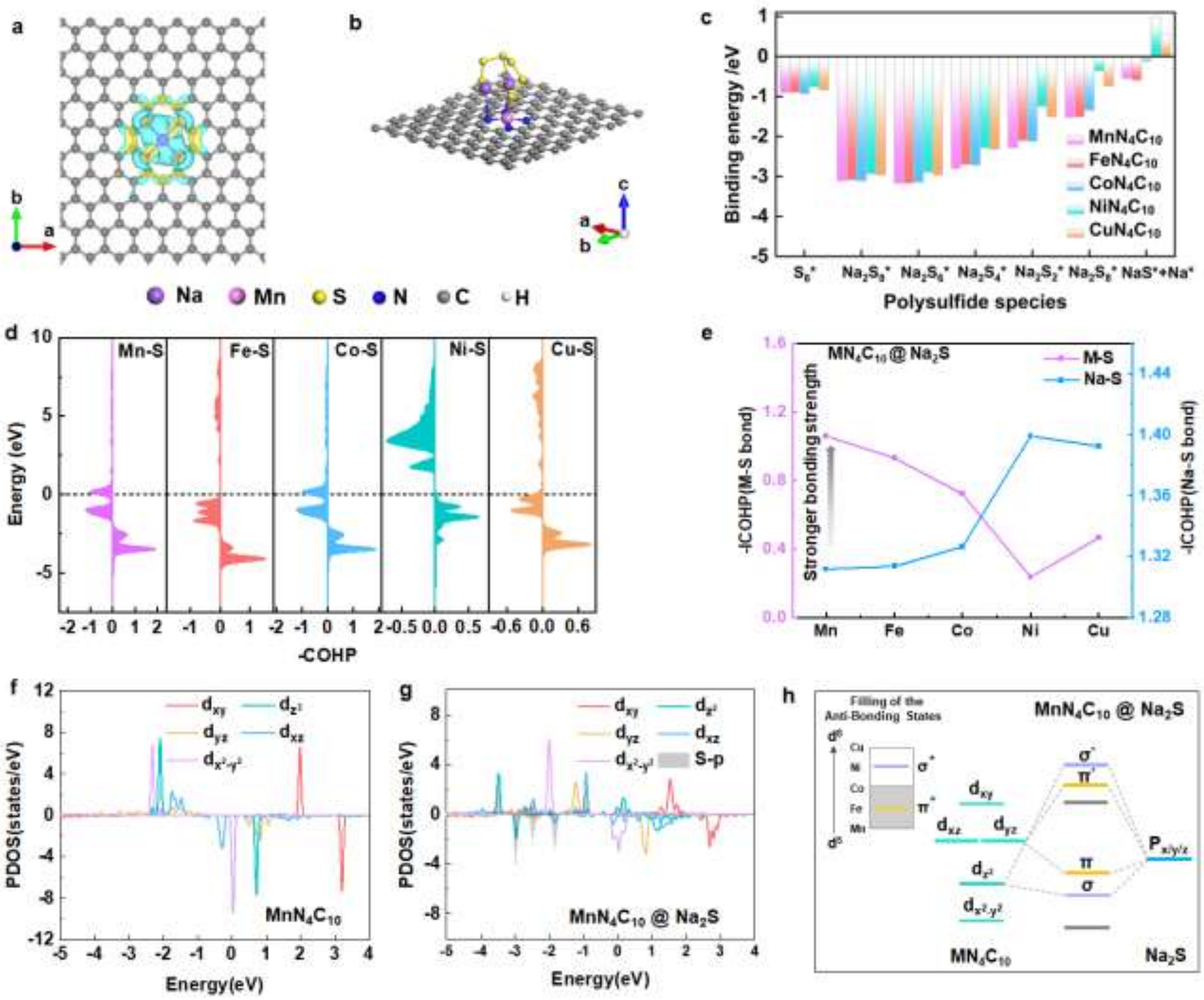

Figure S1. (a) The calculated deformation charge densities MnN_4C_{10}. The iso-surface level is 0.002 eV/Å3. Here, yellow and blue denote the positive area and negative areas, respectively. (b) Optimized configurations of Na_2S_6 on MnN_4C_{10}. (c) The calculated binding energies of NaPS adsorbed on MN_4C_{10}. (d) COHP for six MN_4C_{10} candidates. (e) Strength of Na-S and M-S bond elucidated by the integrated-COHP (-ICOHP) for the MN_4C_{10}@Na_2S adsorption. PDOS of Mn-d orbital of (f) MnN_4C_{10} and (g) MnN_4C_{10}@Na_2S. (h) Scenario of p-d orbital hybridization.

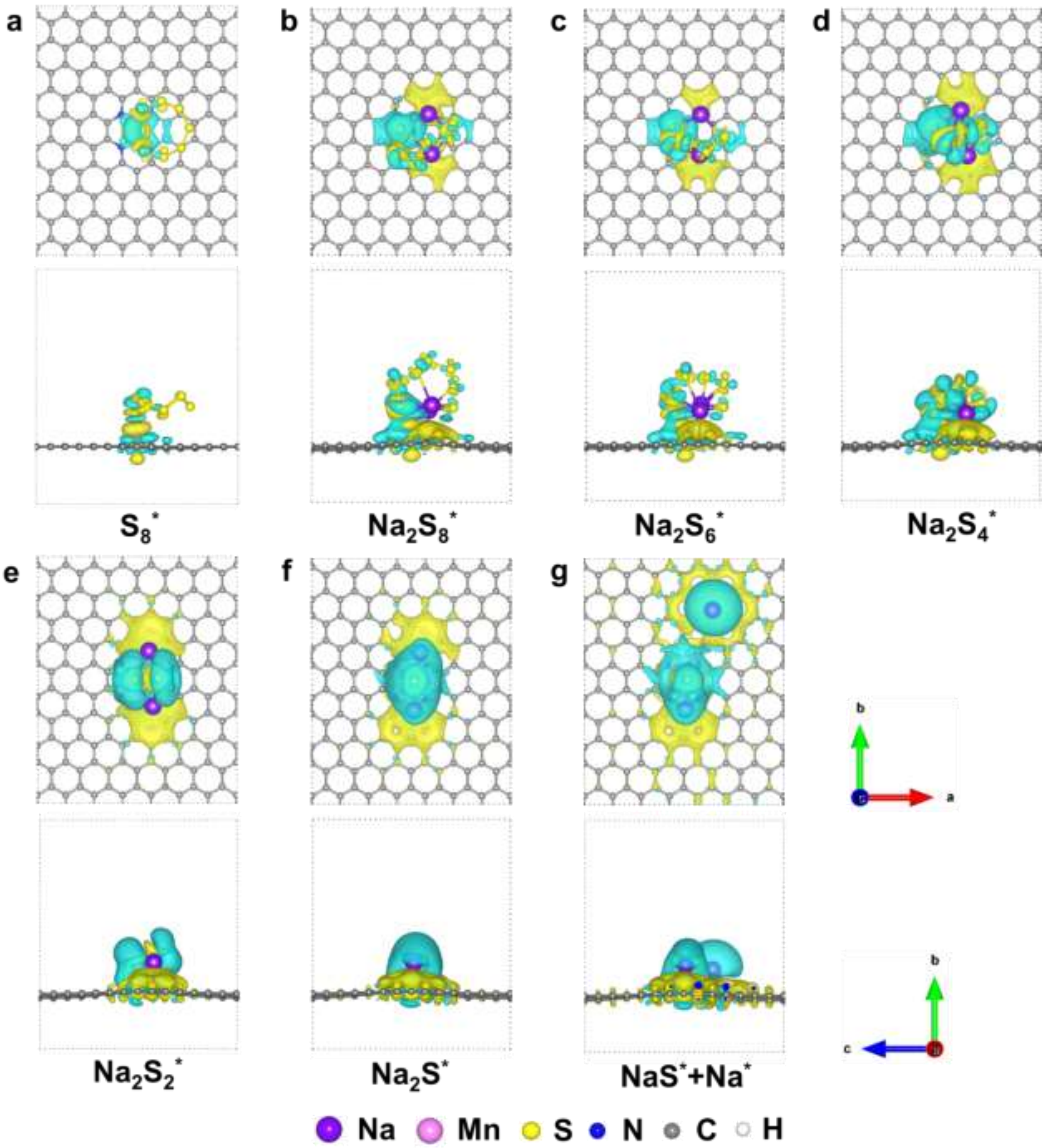

Figure S2. The calculated deformation charge densities of (a) S_8, (b) Na_2S_8, (c) Na_2S_6, (d) Na_2S_4, (e) Na_2S_2, (f)Na_2S, and (g) NaS+S on MnN_4C_{10}. The iso-surface level is 0.004 eV/Å^3. Here, yellow and blue denote the positive area and negative areas, respectively.

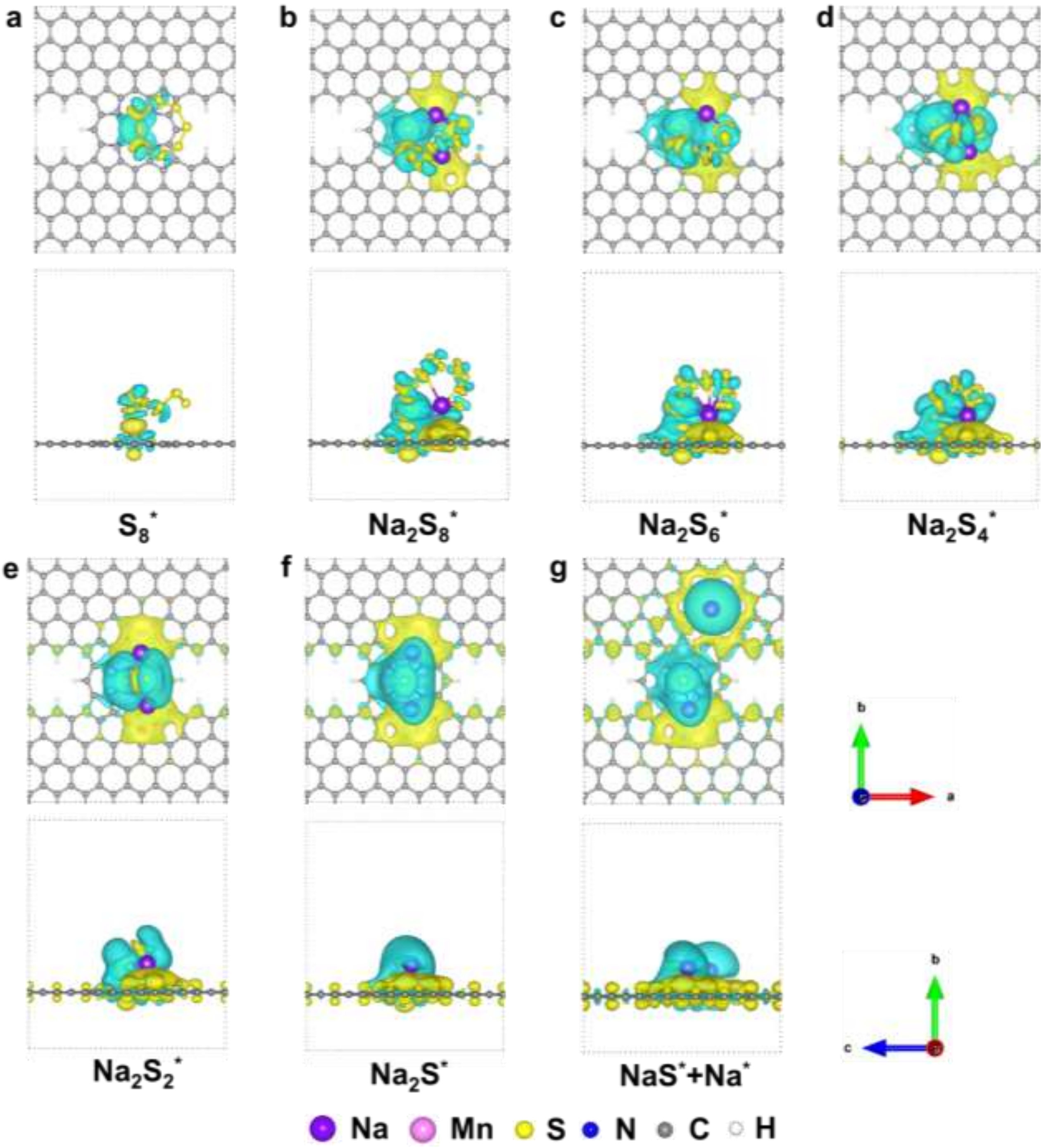

Figure S3. The calculated deformation charge densities of (a) S_8, (b) Na_2S_8, (c) Na_2S_6, (d) Na_2S_4, (e) Na_2S_2, (f)Na_2S, and (g) NaS+S on MnN_4C_{12}. The iso-surface level is 0.004 eV/Å^3. Here, yellow and blue denote the positive area and negative areas, respectively.

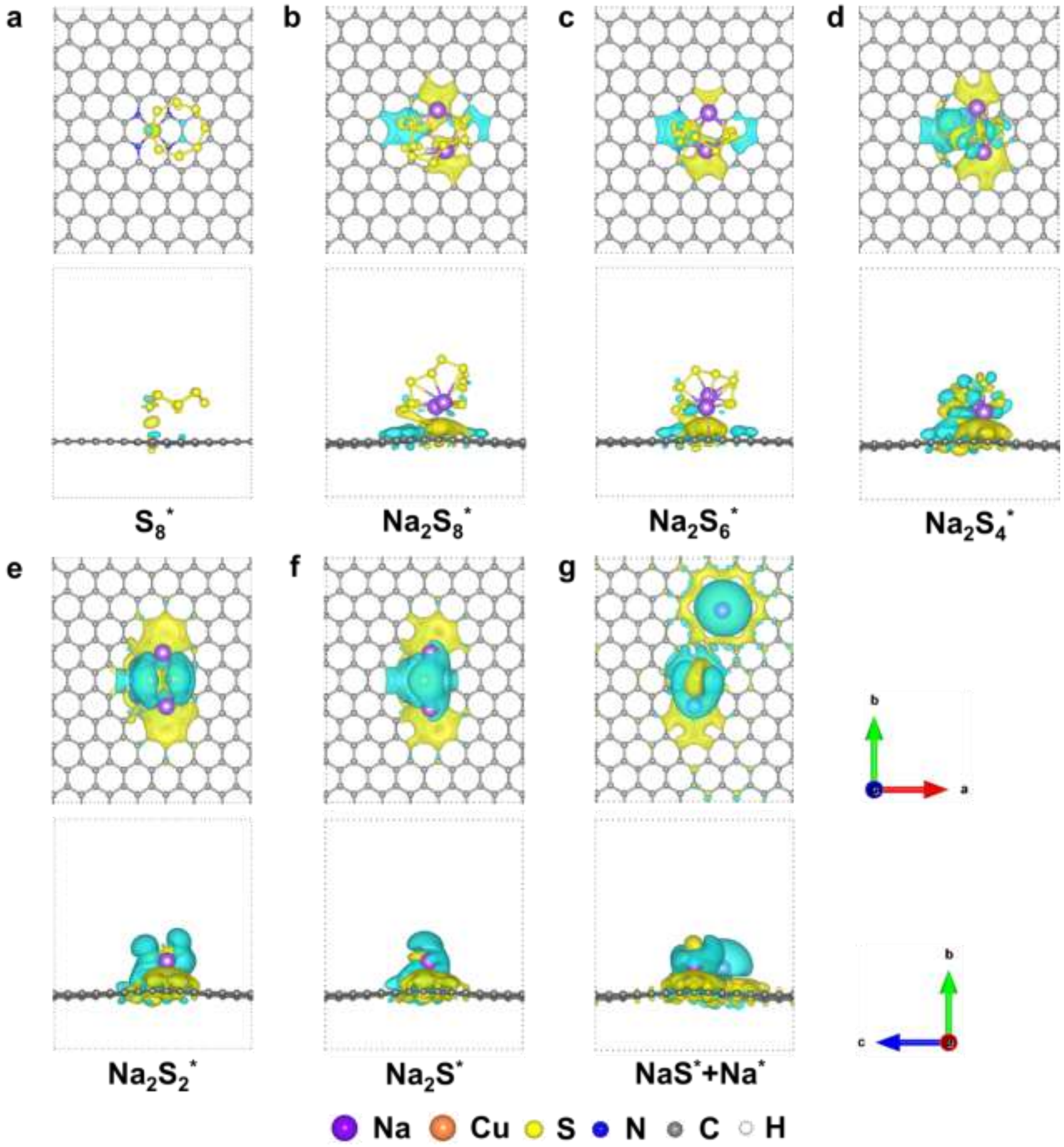

Figure S4. The calculated deformation charge densities of (a) S_8, (b) Na_2S_8, (c) Na_2S_6, (d) Na_2S_4, (e) Na_2S_2, (f)Na_2S, and (g) NaS+S on CuN_4C_{10}. The iso-surface level is 0.004 eV/$Å^3$. Here, yellow and blue denote the positive area and negative areas, respectively.

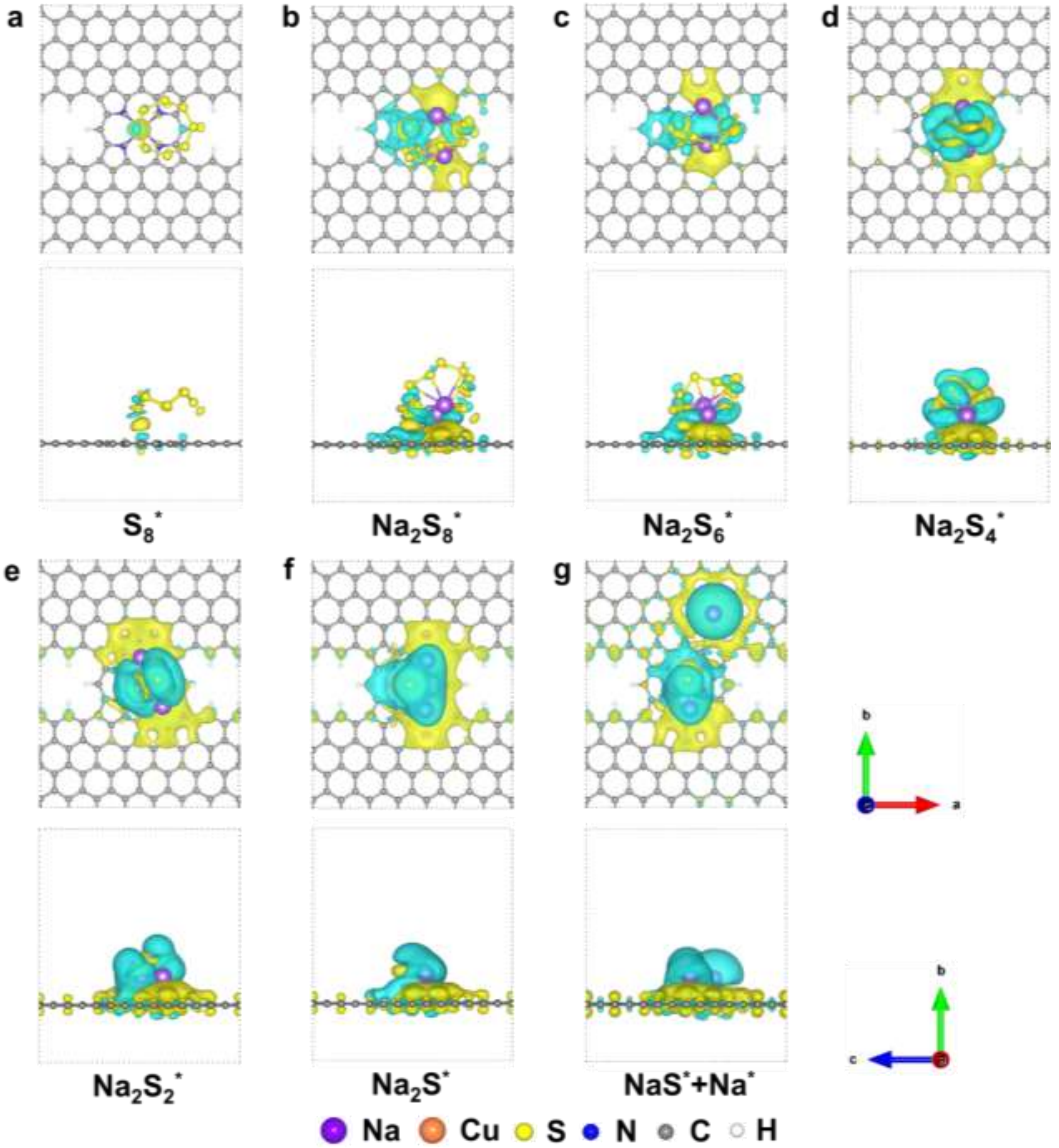

Figure S5. The calculated deformation charge densities of (a) S_8, (b) Na_2S_8, (c) Na_2S_6, (d) Na_2S_4, (e) Na_2S_2, (f)Na_2S, and (g) NaS+S on CuN_4C_{12}. The iso-surface level is 0.004 eV/$Å^3$. Here, yellow and blue denote the positive area and negative areas, respectively.

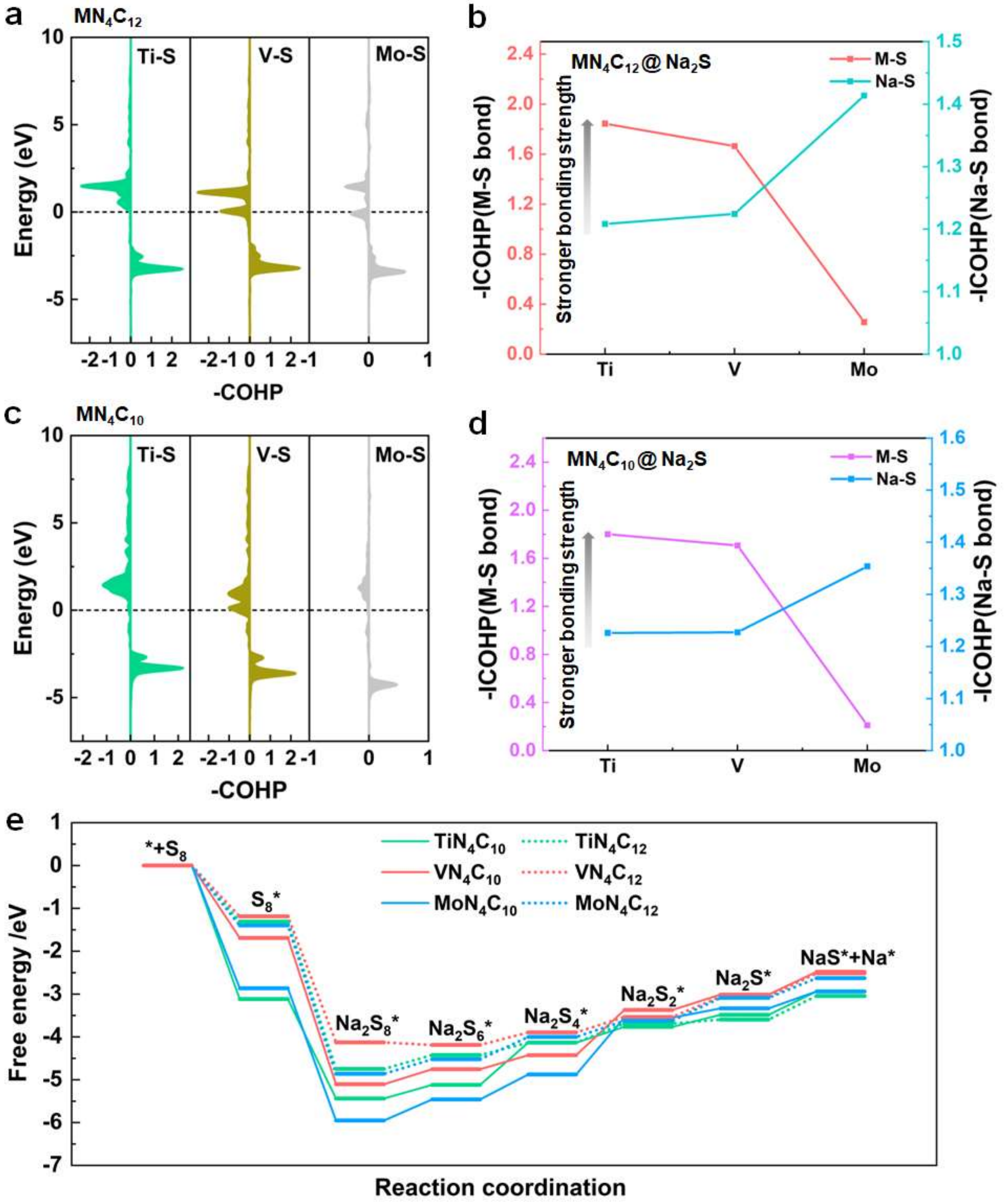

Figure S6. The crystal orbital Hamilton population COHP for (a) MN_4C_{12} and (c) MN_4C_{10}. Strength of Na-S and M-S bond elucidated by the integrated-COHP (-ICOHP) for (b) MN_4C_{12}@Na_2S and (d) MN_4C_{10}@Na_2S adsorption. (e)The calculated Gibbs free energy profiles for the reduction of NaPS and the Na2S decomposition on MN_4 (M = Ti, V, and Mo).

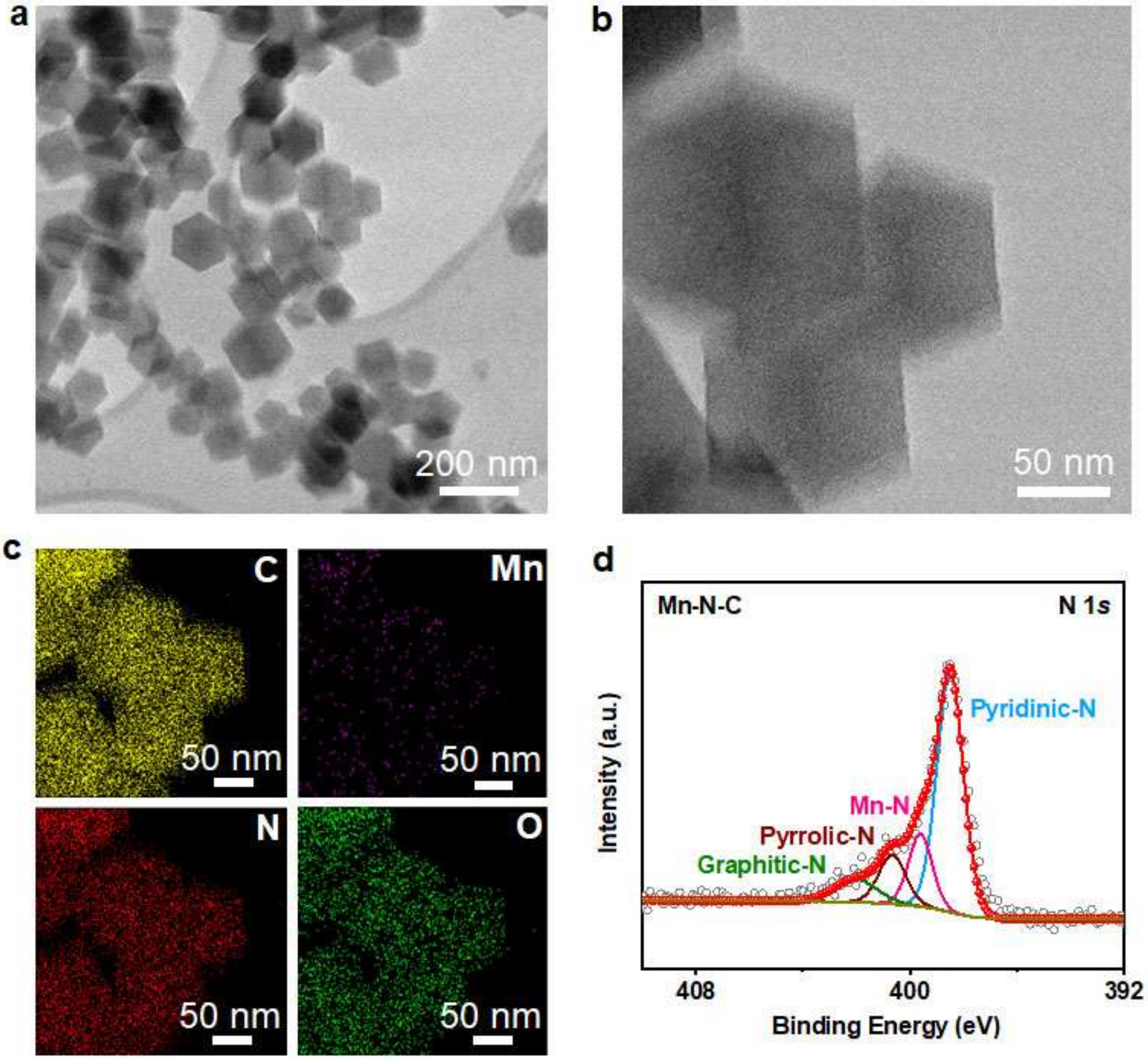

Figure S7. (a) Low-magnification TEM image, (b) high-magnification TEM image, (c) element mapping images and (d) N 1*s* XPS spectra for Mn-N-C SACs.

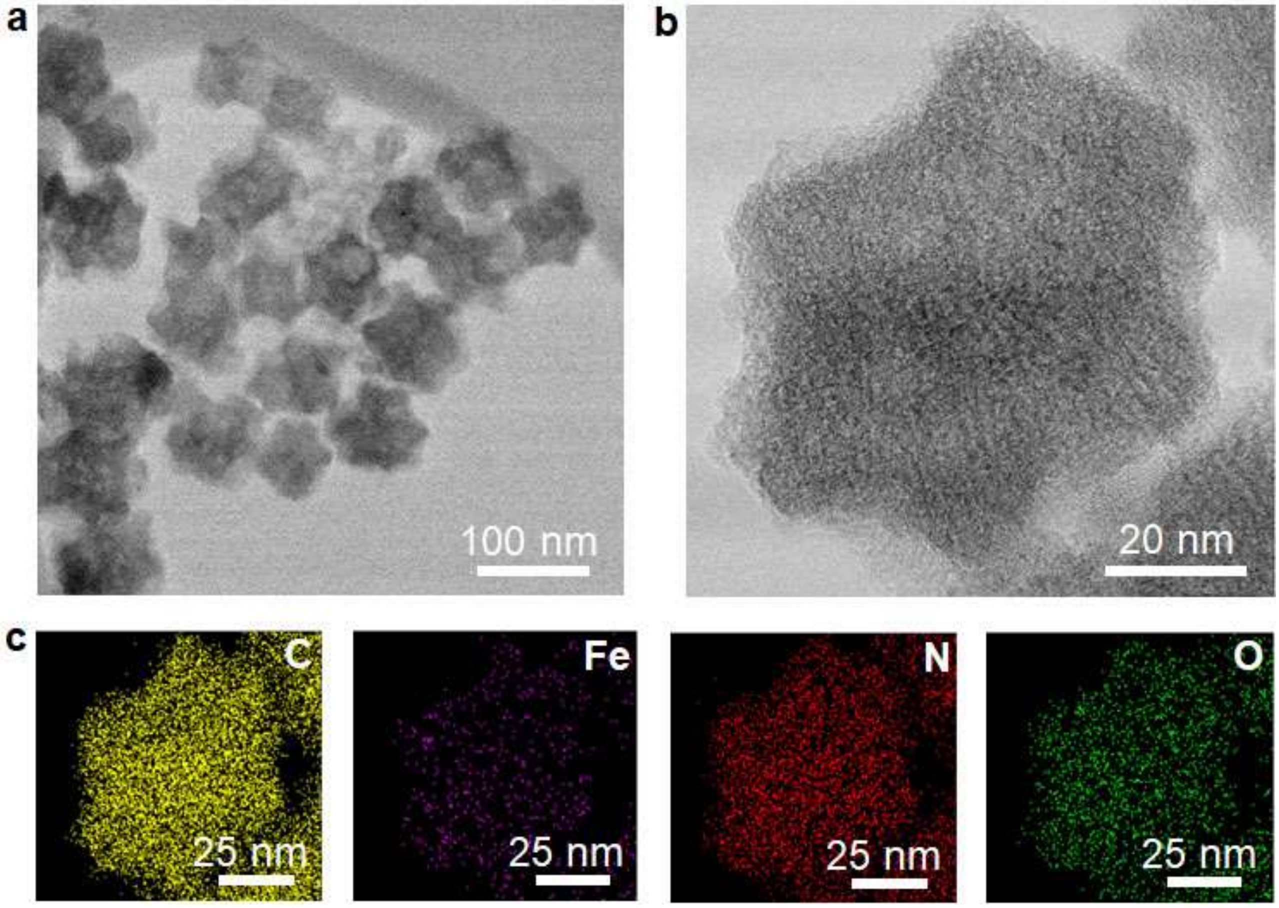

Figure S8. (a) Low-magnification TEM image, (b) High-magnification TEM image and (c) element mapping images of Fe-N-C SACs.

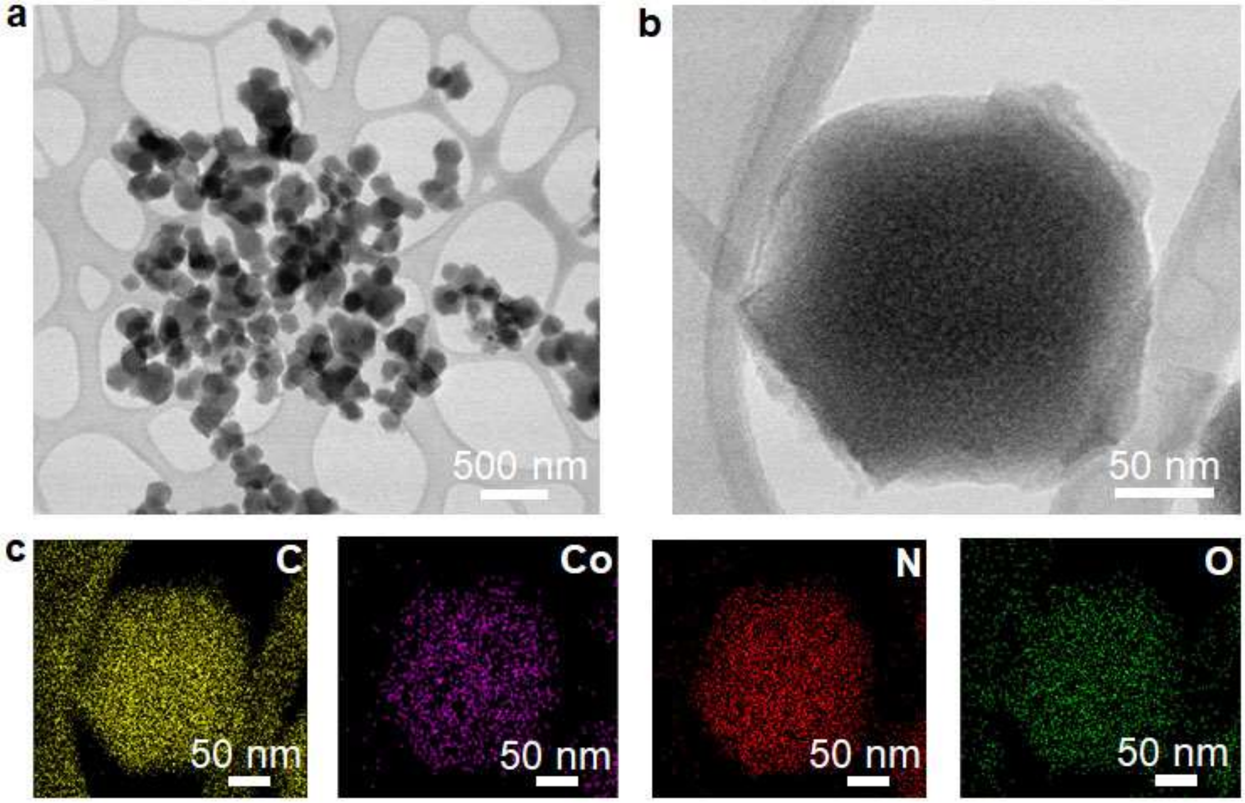

Figure S9. (a) Low-magnification TEM image, (b) High-magnification TEM image and (c) element mapping images of Co-N-C SACs.

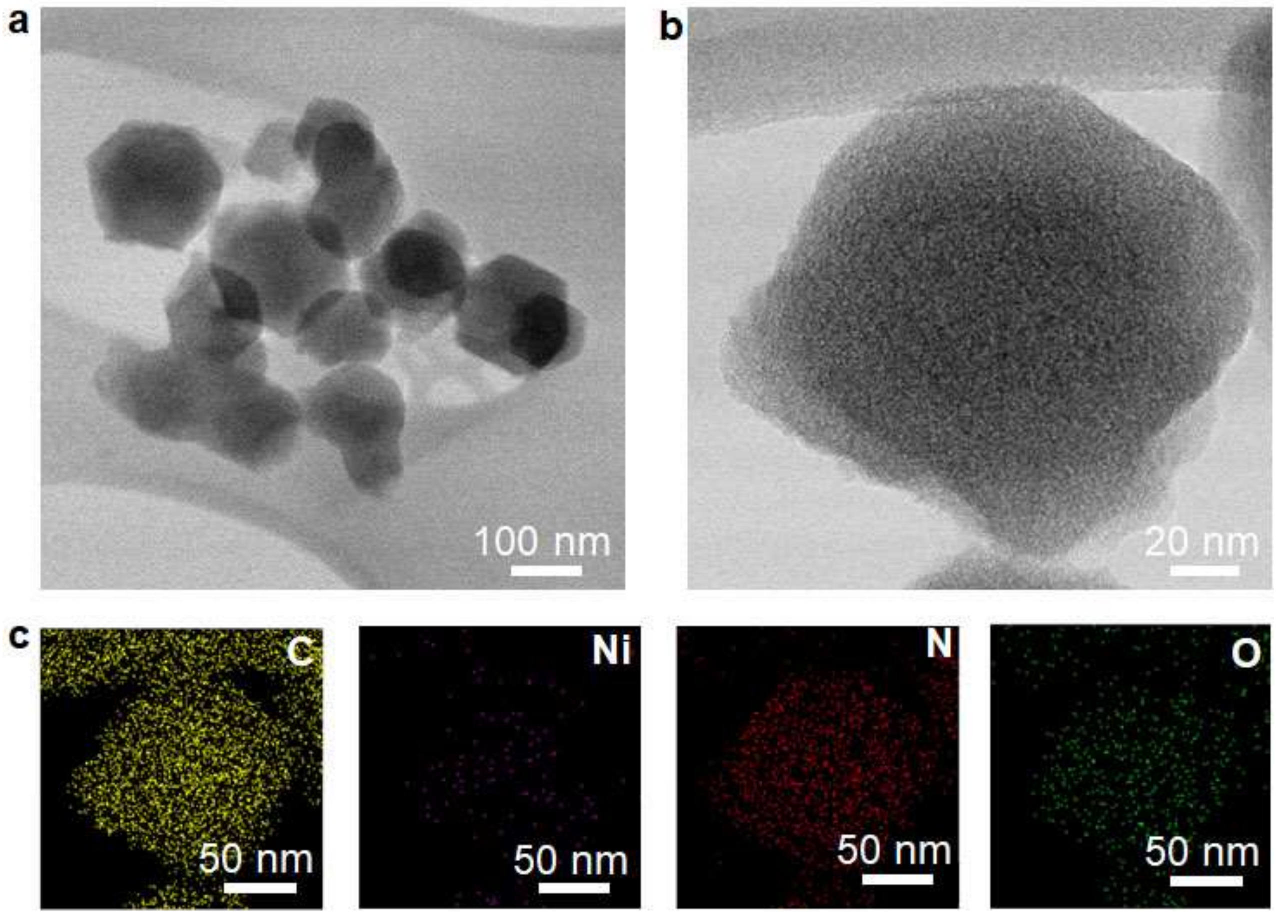

Figure S10. (a) Low-magnification TEM image, (b) High-magnification TEM image and (c) element mapping images of Ni-N-C SACs.

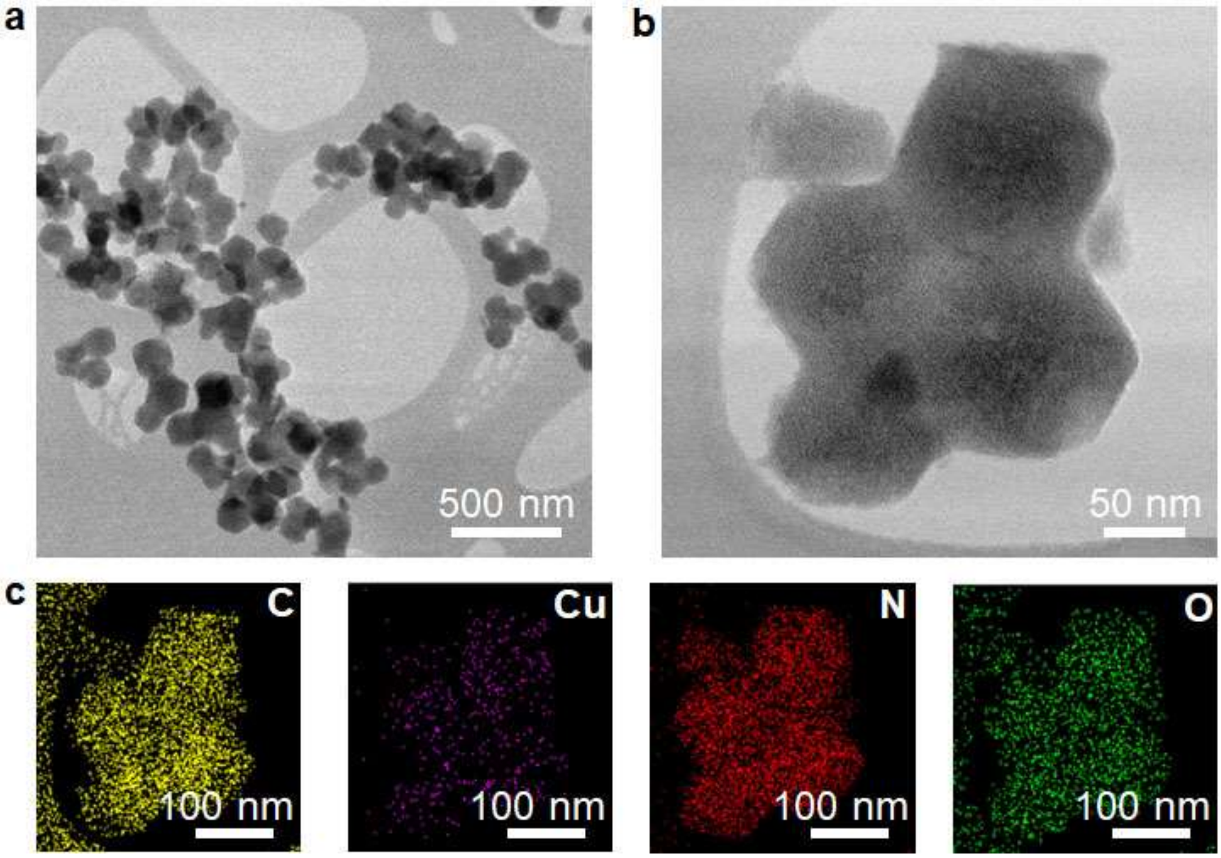

Figure S11. (a) Low-magnification TEM image, (b) High-magnification TEM image and (c) element mapping images of Cu-N-C SACs.

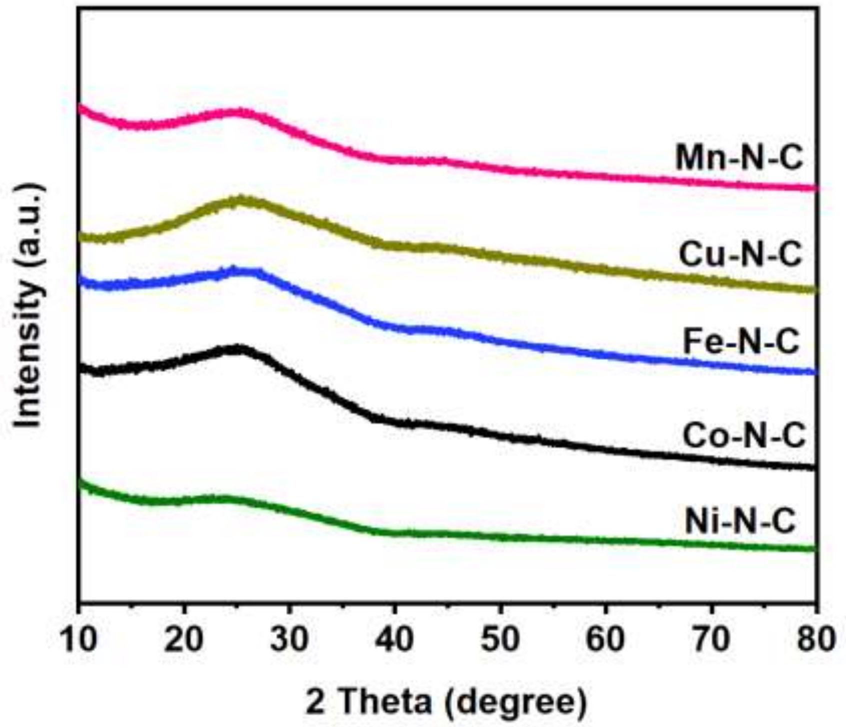

Figure S12. XRD patterns of Mn-N-C, Cu-N-C, Fe-N-C, Co-N-C and Ni-N-C SACs.

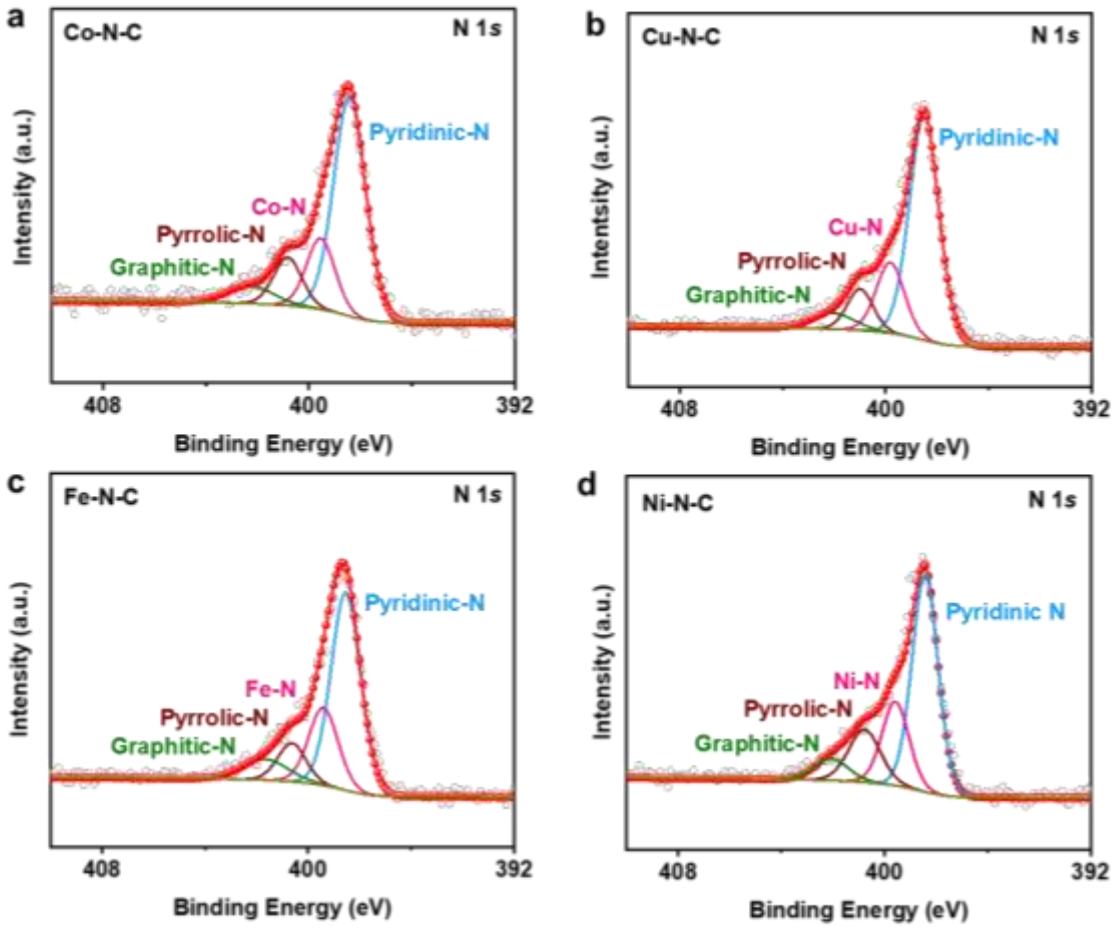

Figure S13. N 1*s* XPS spectra of (a) Co-N-C, (b) Cu-N-C, (c) Fe-N-C and (d) Ni-N-C SACs.

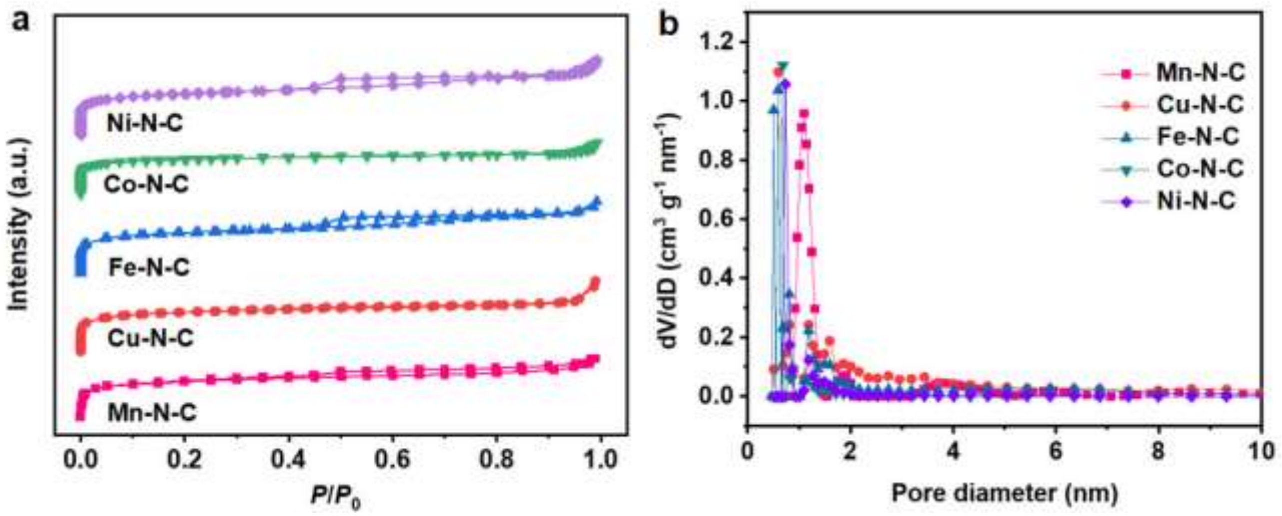

Figure S14. a) N_2 adsorption–desorption isotherms and b) the corresponding pore size distribution of of Mn-N-C, Co-N-C, Ni-N-C, Cu-N-C and Fe-N-C SACs.

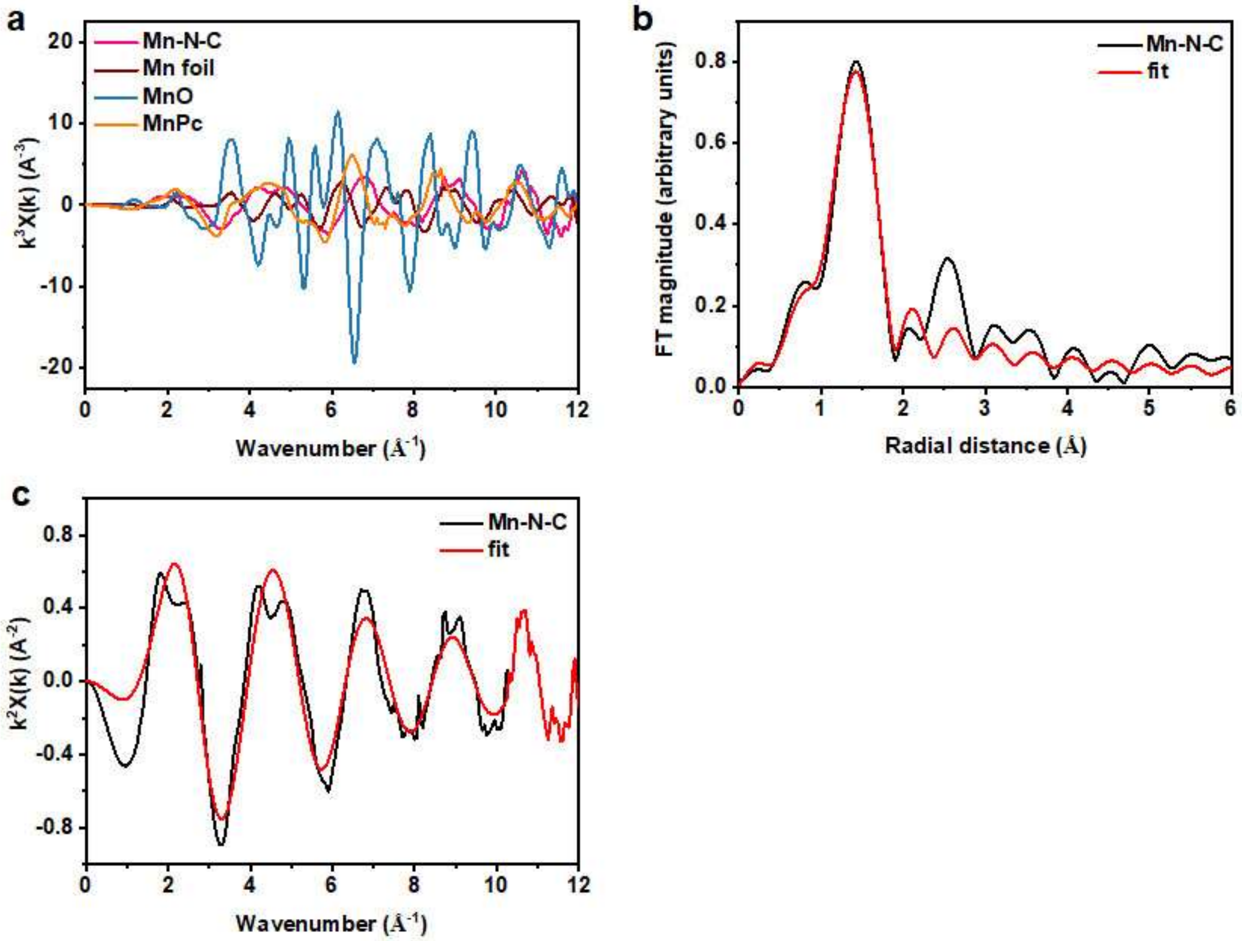

Figure S15. (a) Fourier-transform EXAFS spectra in *k*-space of Mn-N-C SACs, Mn foil, MnO and MnPc at manganese K-edge. The corresponding FT-EXAFS fitting of manganese K-edge in *R*-space (b) and *k*-space (c) of Mn-N-C SACs at manganese K-edge.

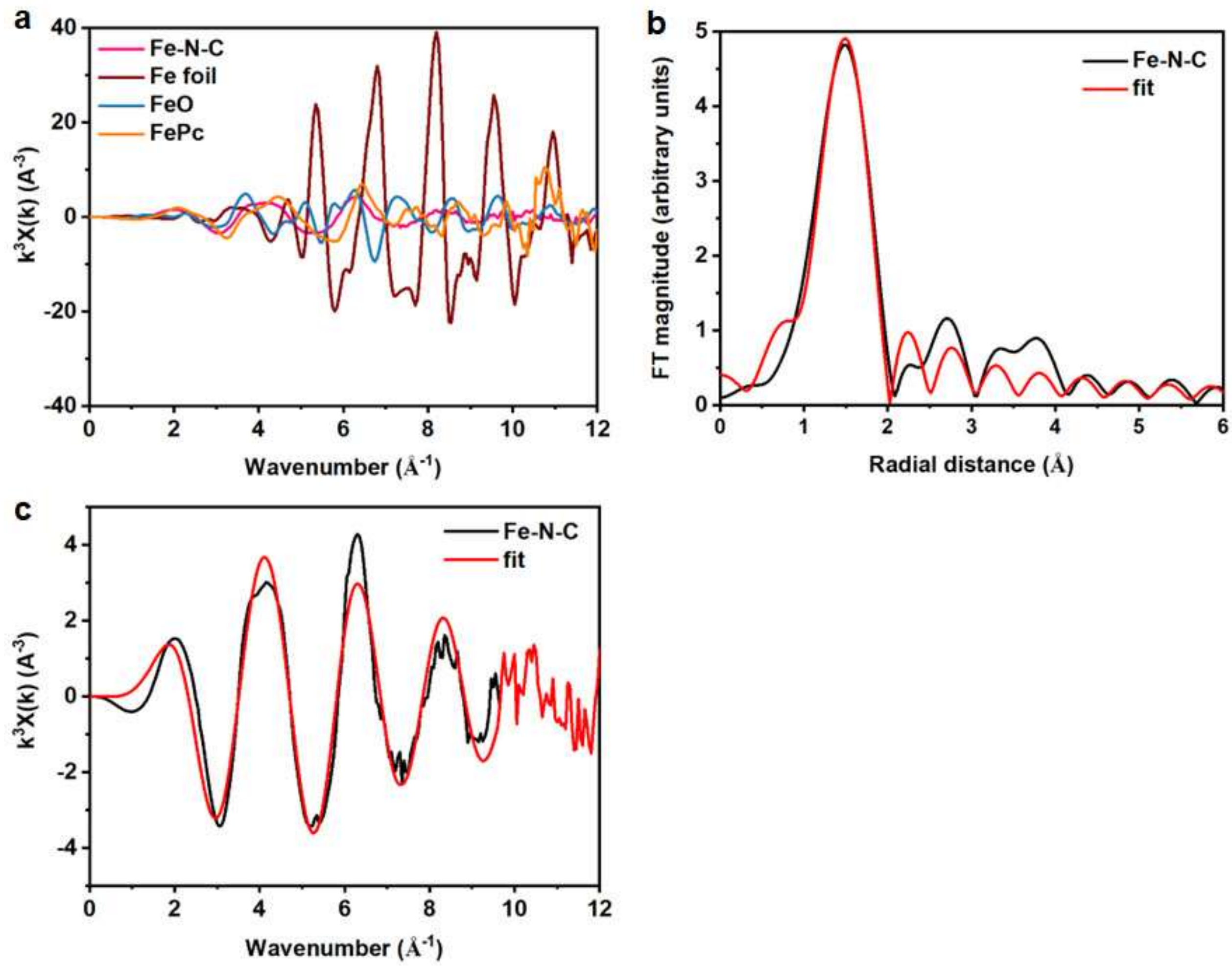

Figure S16. (a) Fourier-transform EXAFS spectra in *k*-space of Fe-N-C, Fe foil, FeO and FePc at iron K-edge. The corresponding FT-EXAFS fitting of iron K-edge in *R*-space (b) and *k*-space (c) of Fe-N-C SACs at iron K-edge.

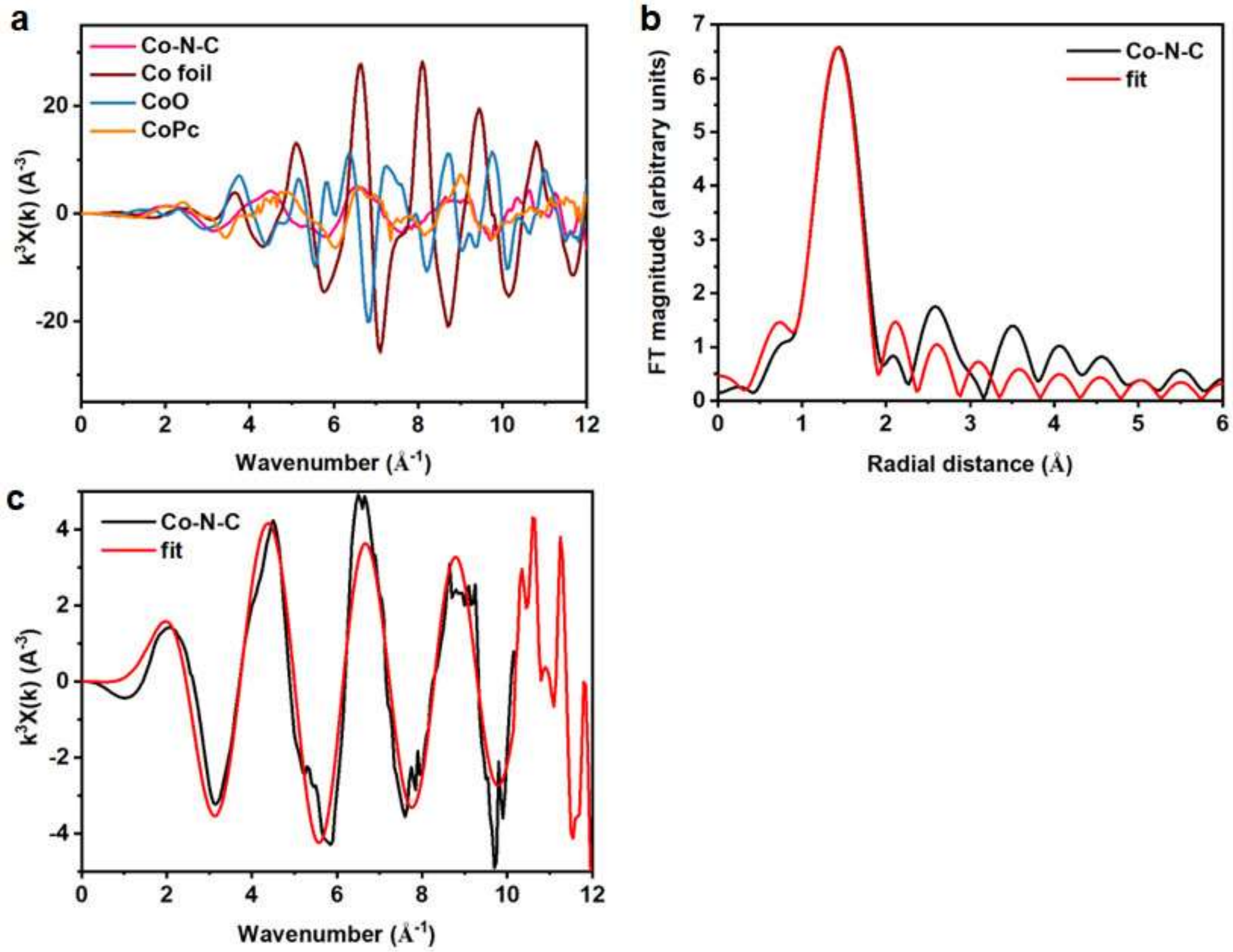

Figure S17. (a) Fourier-transform EXAFS spectra in *k*-space of Co-N-C, Co foil, CoO and CoPc at cobalt K-edge. The corresponding FT-EXAFS fitting of iron K-edge in *R*-space (b) and *k*-space (c) of Co-N-C SACs at cobalt K-edge.

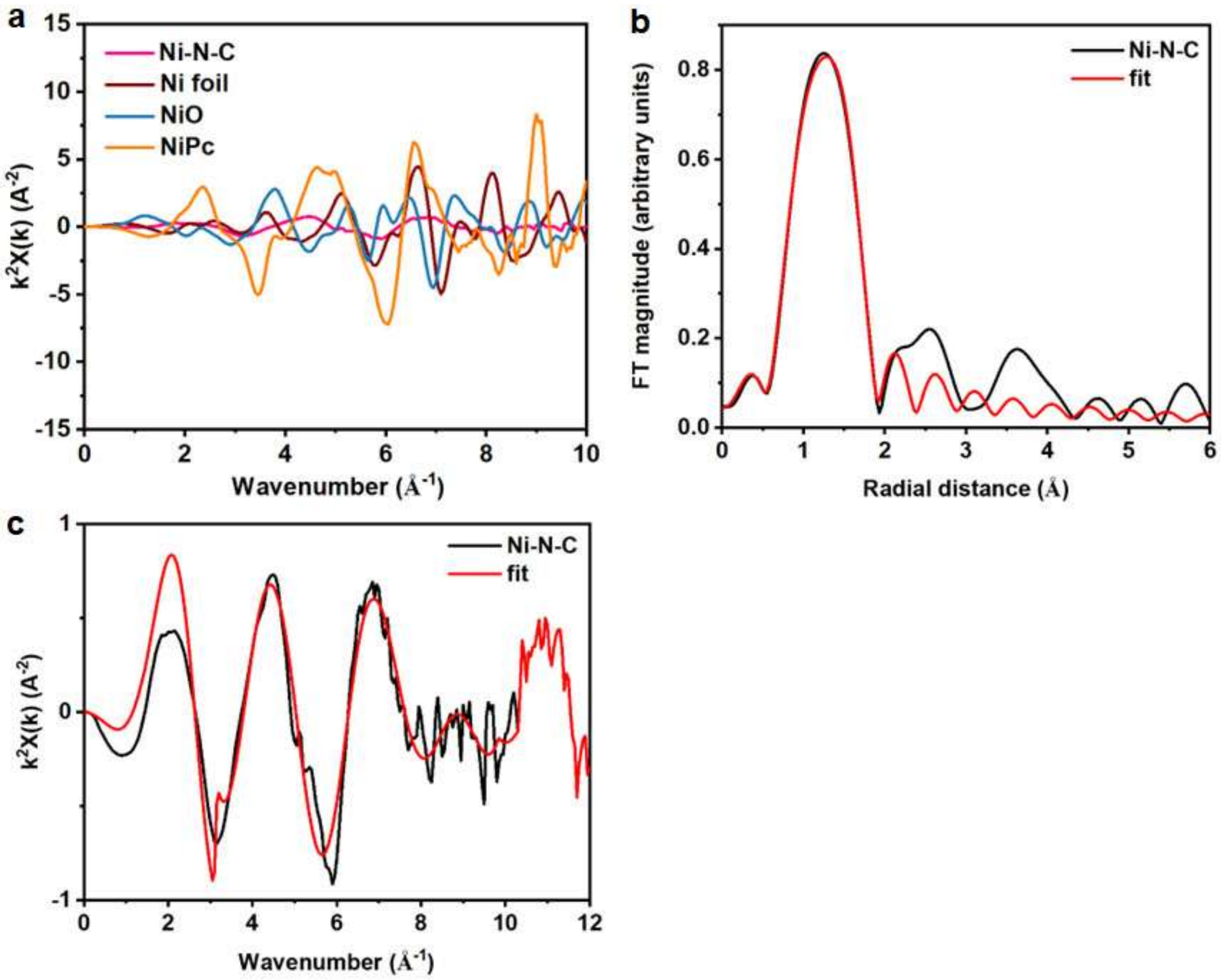

Figure S18. (a) Fourier-transform EXAFS spectra in *k*-space of Ni-N-C, Ni foil, NiO and NiPc at nickel K-edge. The corresponding FT-EXAFS fitting of nickel K-edge in *R*-space (b) and *k*-space (c) of Ni-N-C SACs at nickel K-edge.

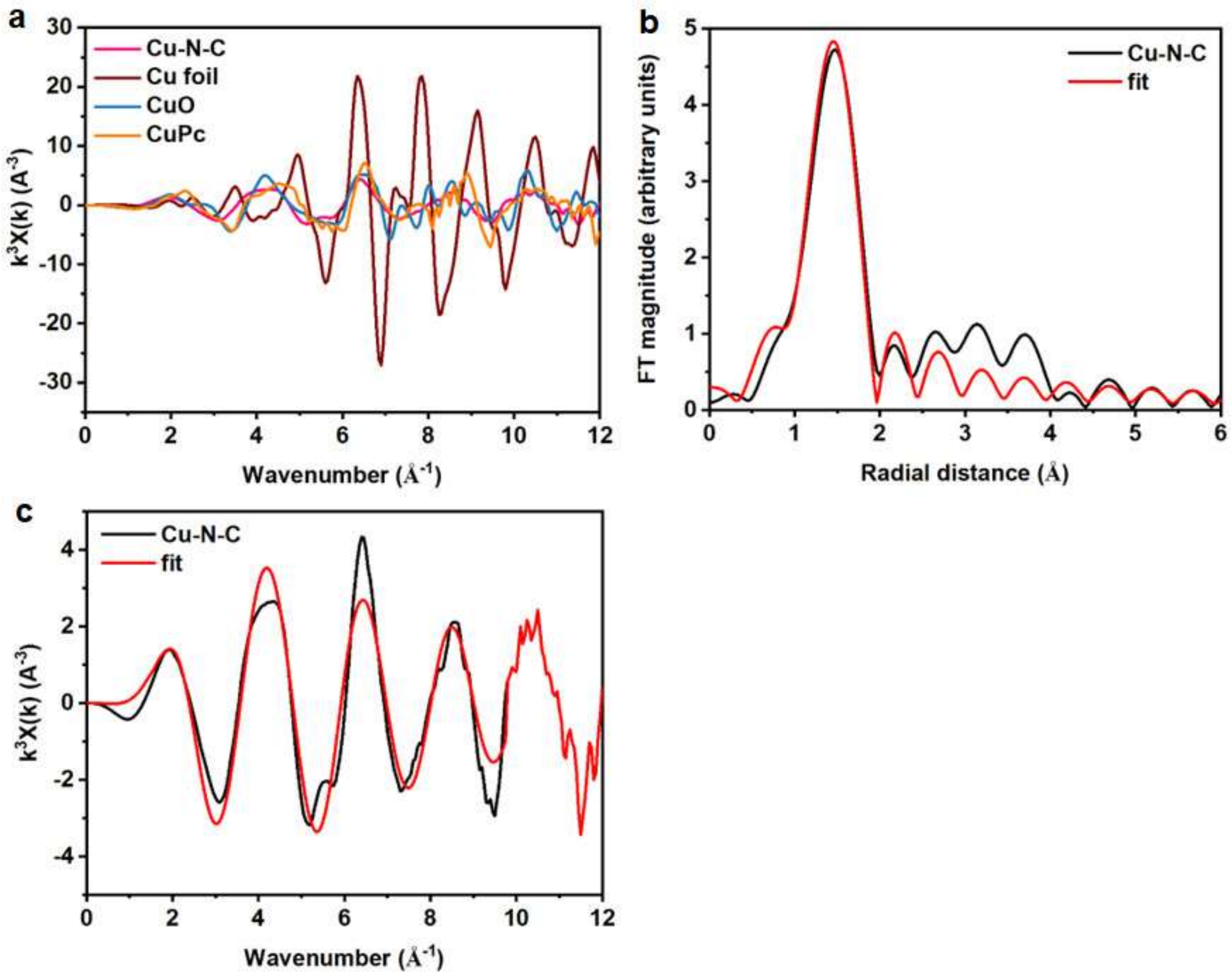

Figure S19. (a) Fourier-transform EXAFS spectra in *k*-space of Cu-N-C, Cu foil, CuO and CuPc at copper K-edge. The corresponding FT-EXAFS fitting of copper K-edge in *R*-space (b) and *k*-space (c) of Cu-N-C SACs at copper K-edge.

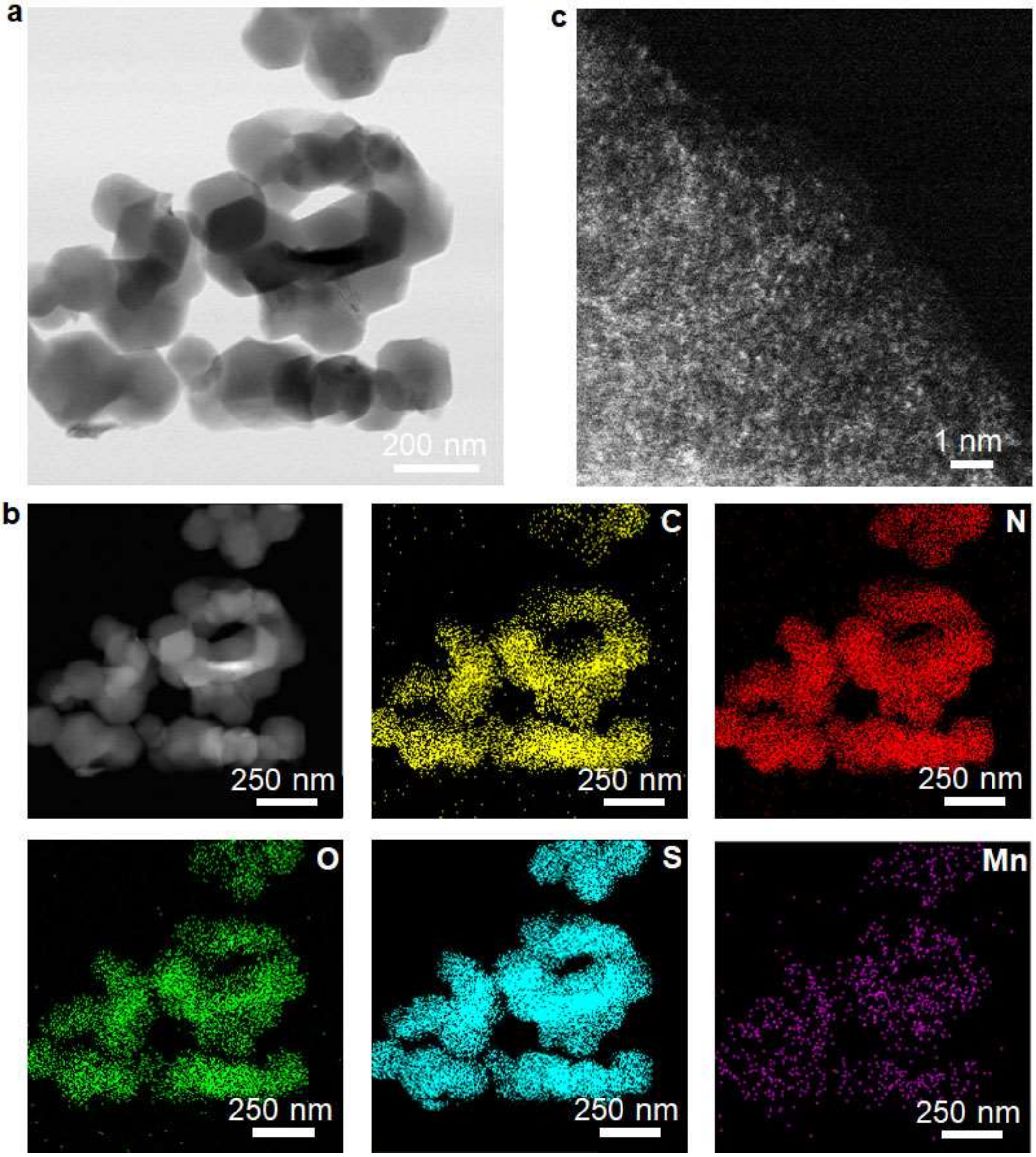

Figure S20. (a) Low-magnification TEM image, (b) element mapping images and (c) aberration-corrected HAADF-STEM image of S@Mn–N–C materials.

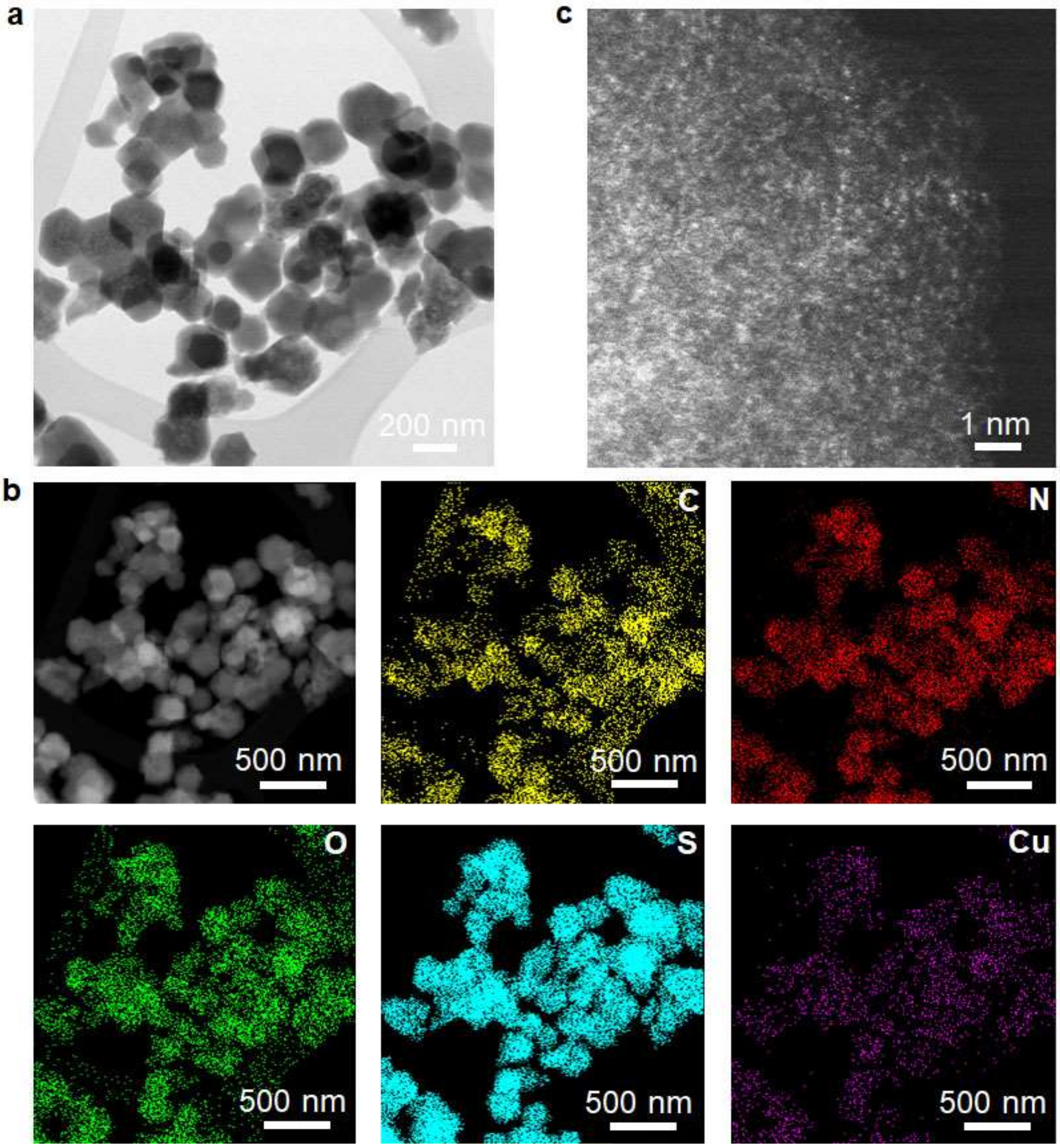

Figure S21. (a) Low-magnification TEM image, (b) element mapping images and (c) aberration-corrected HAADF-STEM image of S@Cu–N–C materials.

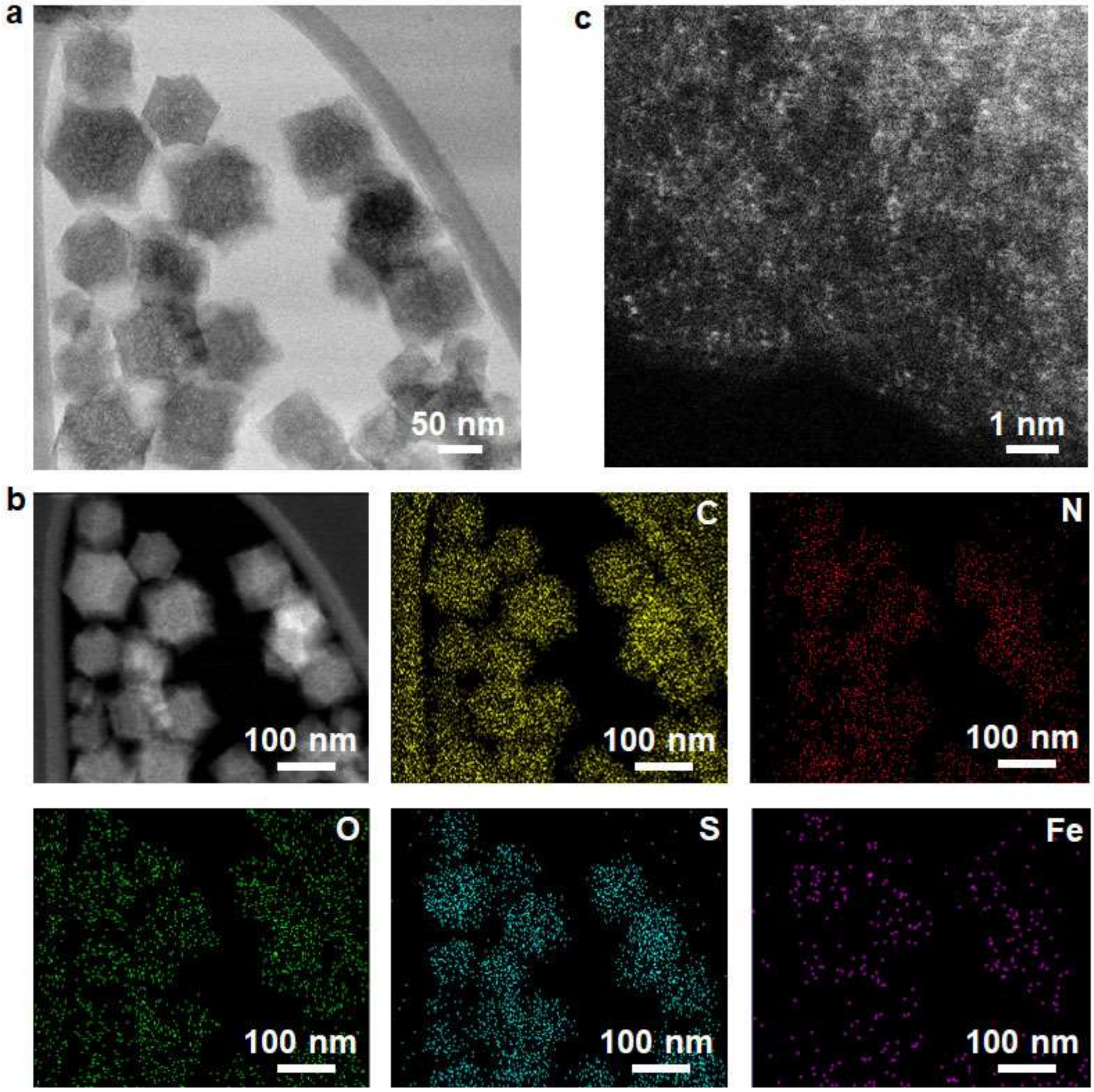

Figure S22. (a) Low-magnification TEM image, (b) element mapping images and (c) aberration-corrected HAADF-STEM image of S@Fe–N–C materials.

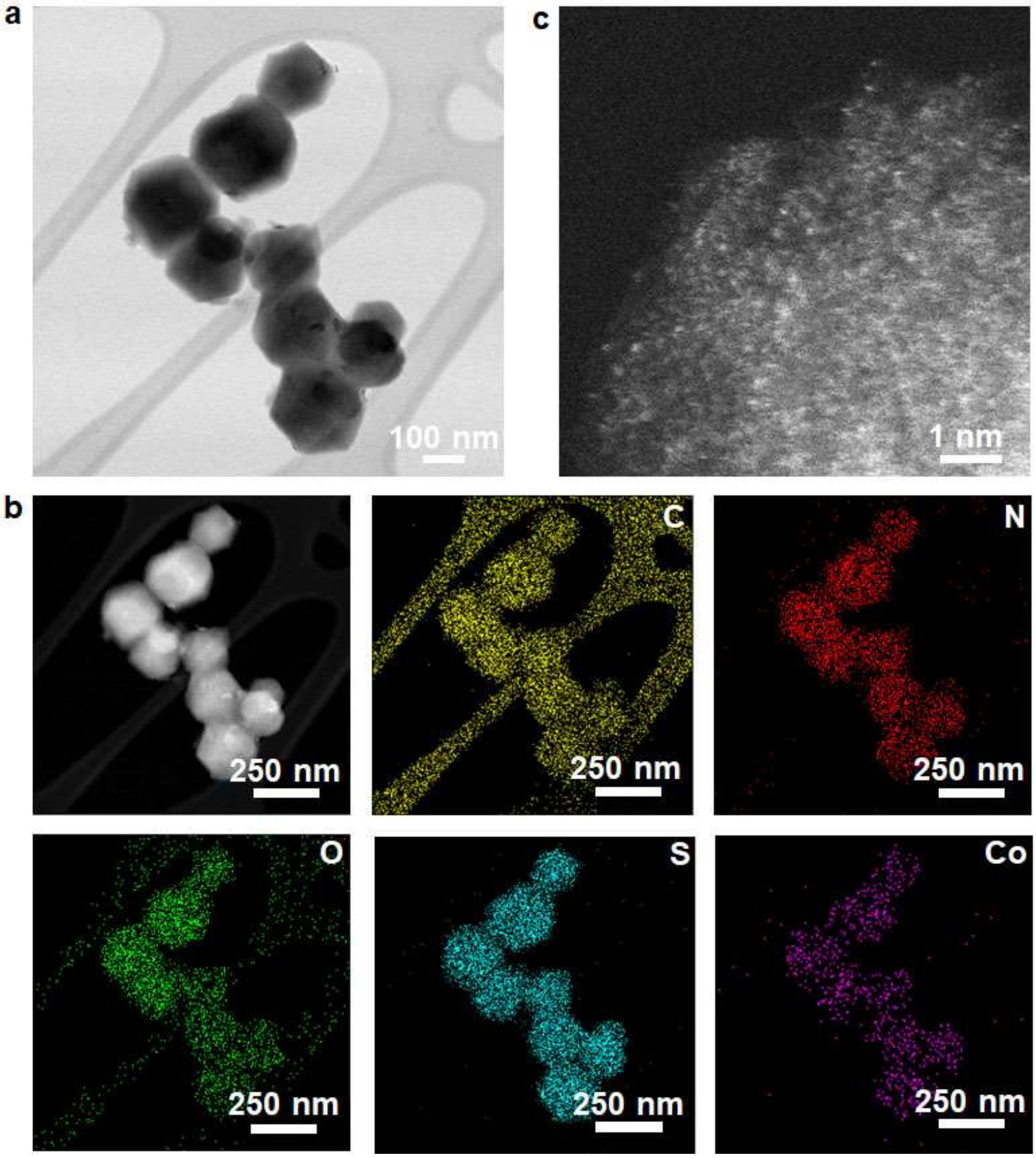

Figure S23. (a) Low-magnification TEM image, (b) element mapping images and (c) aberration-corrected HAADF-STEM image of S@Co–N–C materials.

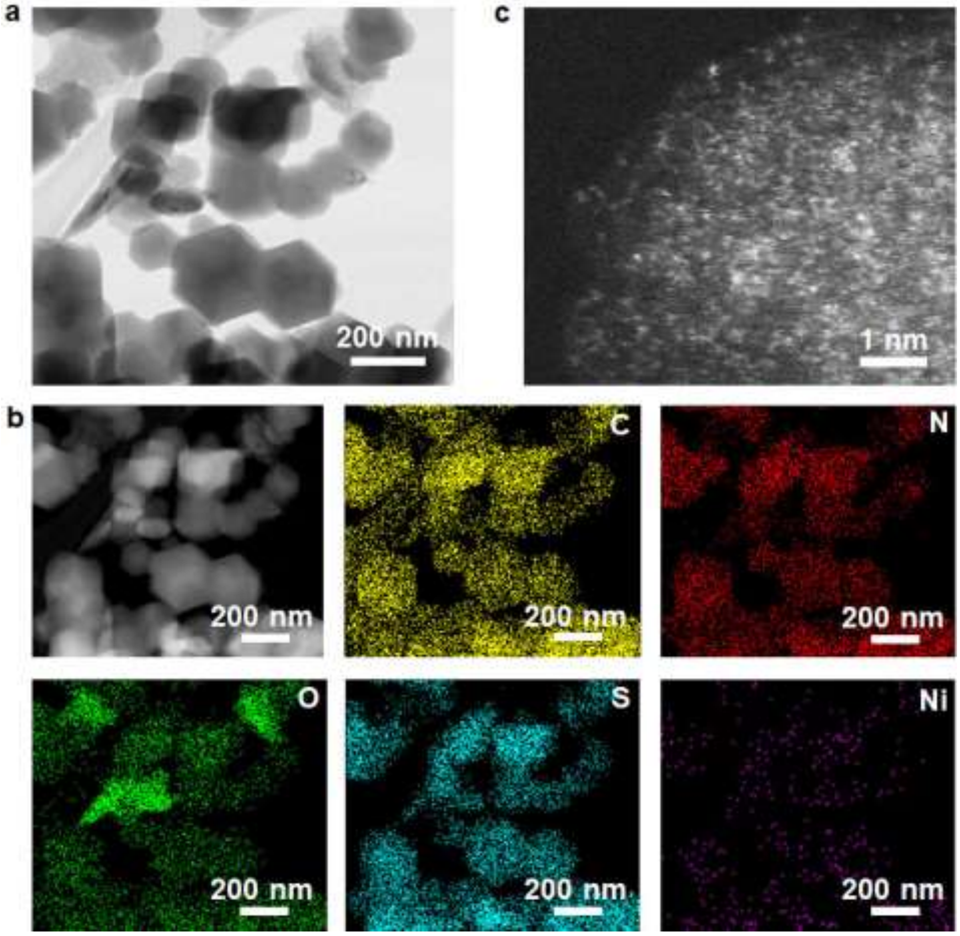

Figure S24. (a) Low-magnification TEM image, (b) element mapping images and (c) aberration-corrected HAADF-STEM image of S@Ni–N–C materials.

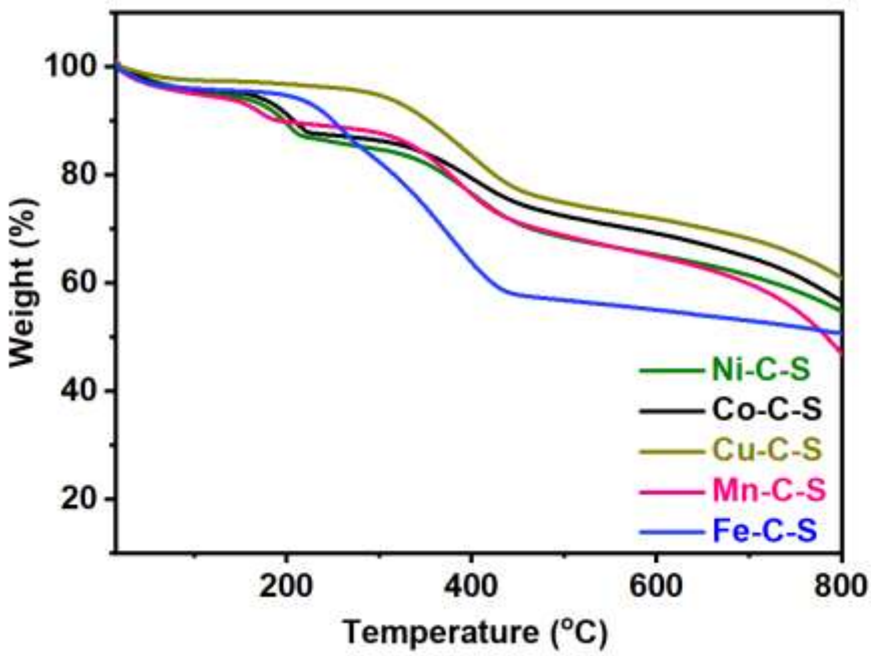

Figure S25. TGA curves of S@Mn-N-C, S@Cu-N-C, S@Fe-N-C, S@Co-N-C and S@Ni-N-C materials.

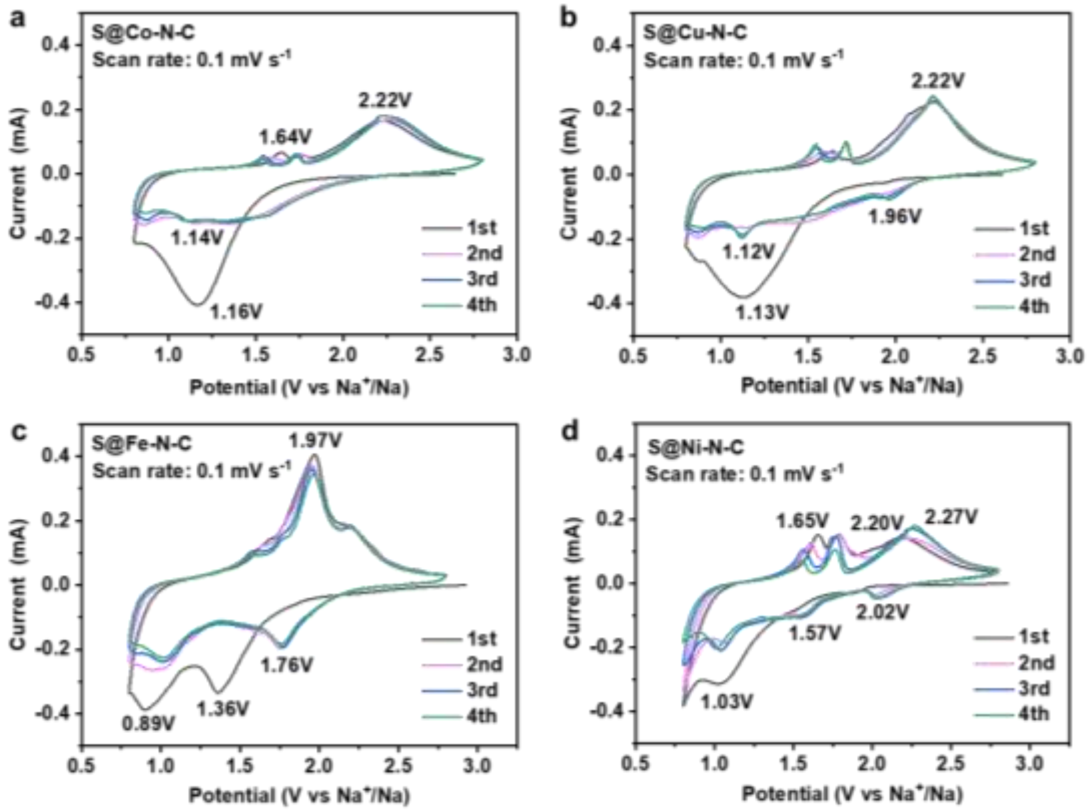

Figure S26. CV curves of (a) S@Co-N-C, (b) S@Cu-N-C, (c) S@Fe-N-C and (d) S@Ni-N-C electrodes at a scan rate of 0.1 mV s^{-1} (the first four cycles).

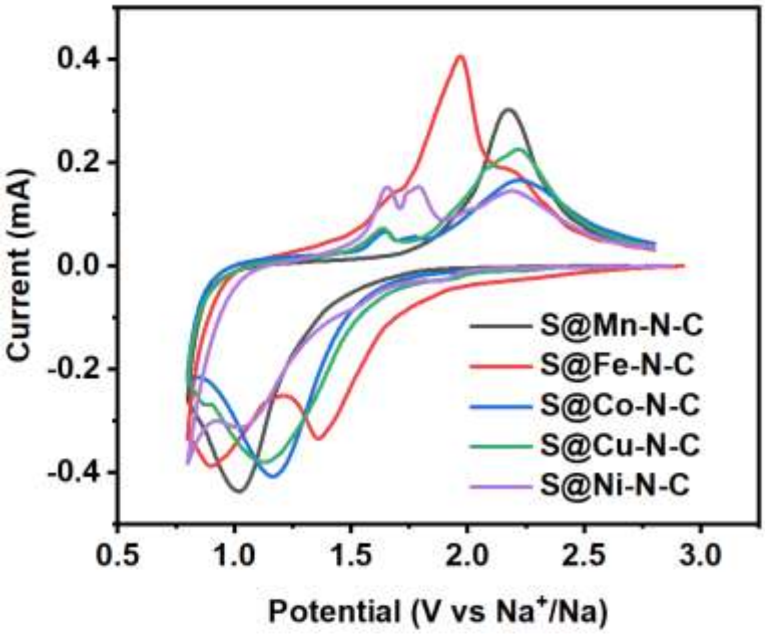

Figure S27. CV curves of S@Mn-N-C, S@Cu-N-C, S@Fe-N-C, S@Co-N-C and S@Ni-N-C electrodes for the first cycle at a scan rate of 0.1 mV s^{-1}.

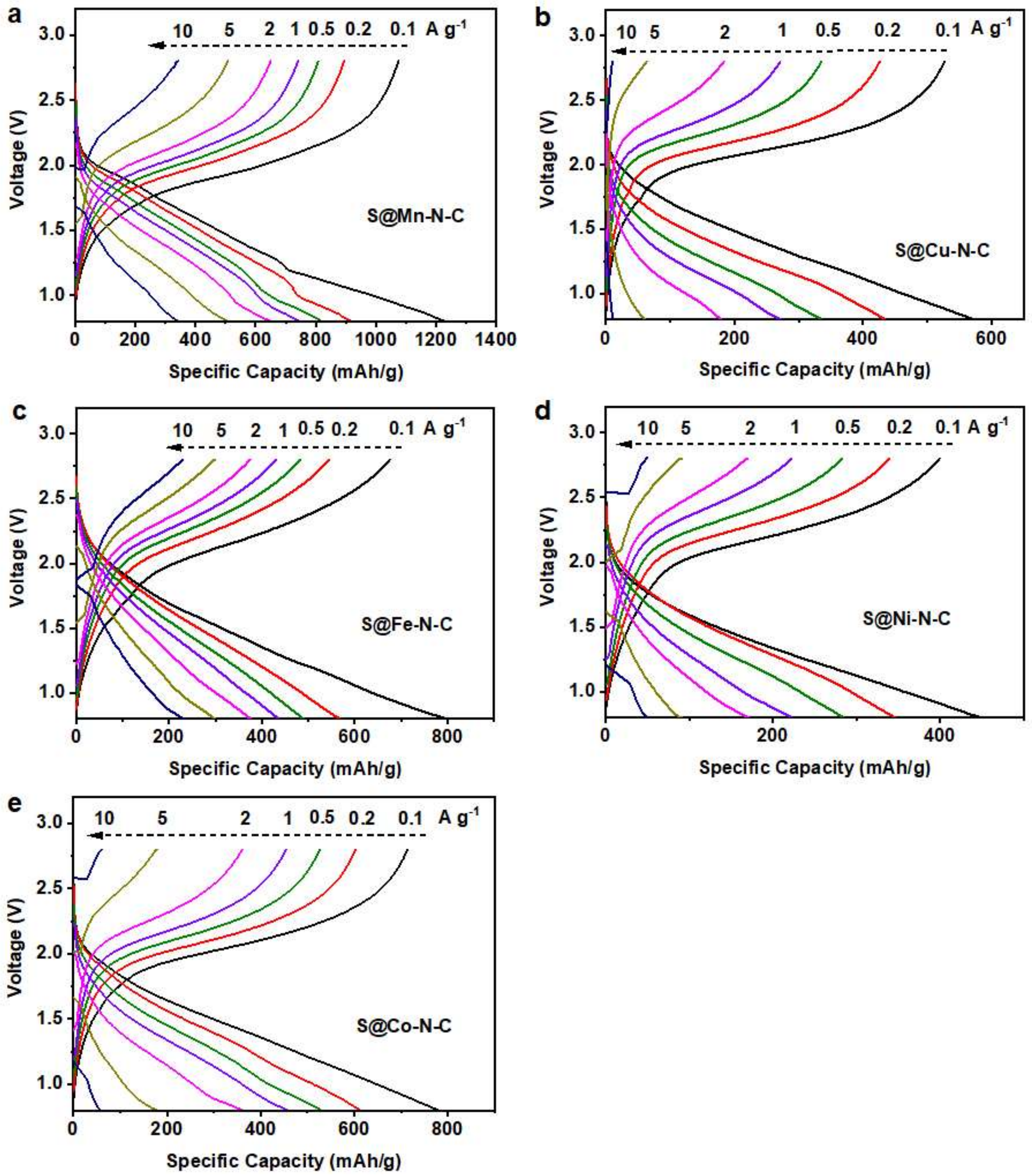

Figure S28. The discharge-charge voltage profiles of (a) S@Mn-N-C, (b) S@Cu-N-C, (c) S@Fe-N-C, (d) S@Ni-N-C and (a) S@Co-N-C electrodes at different current densities.

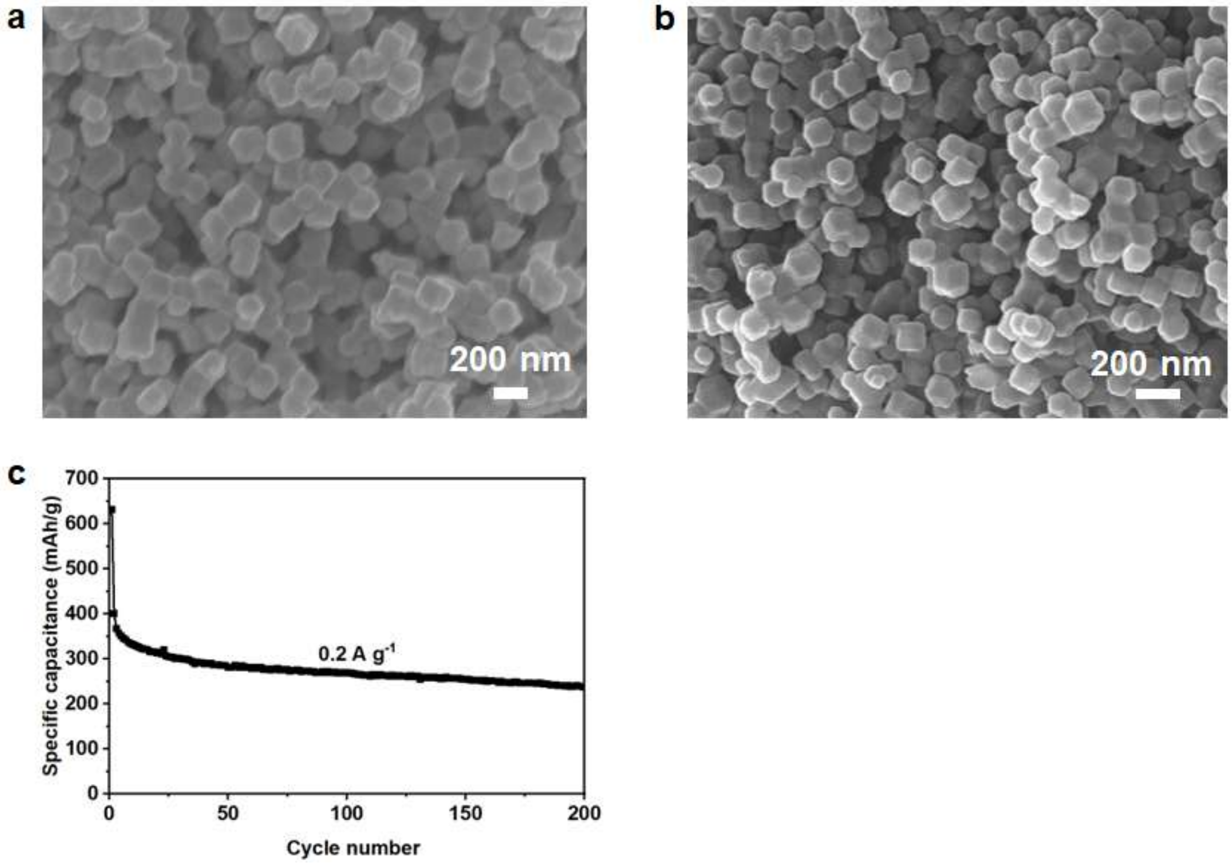

Figure S29. (a) SEM image of ZIF-8, (b) SEM image of N-doped carbon derived from ZIF-8, (c) Cycling performance of S@N-C electrode at 0.2 A/g for 200 cycles.

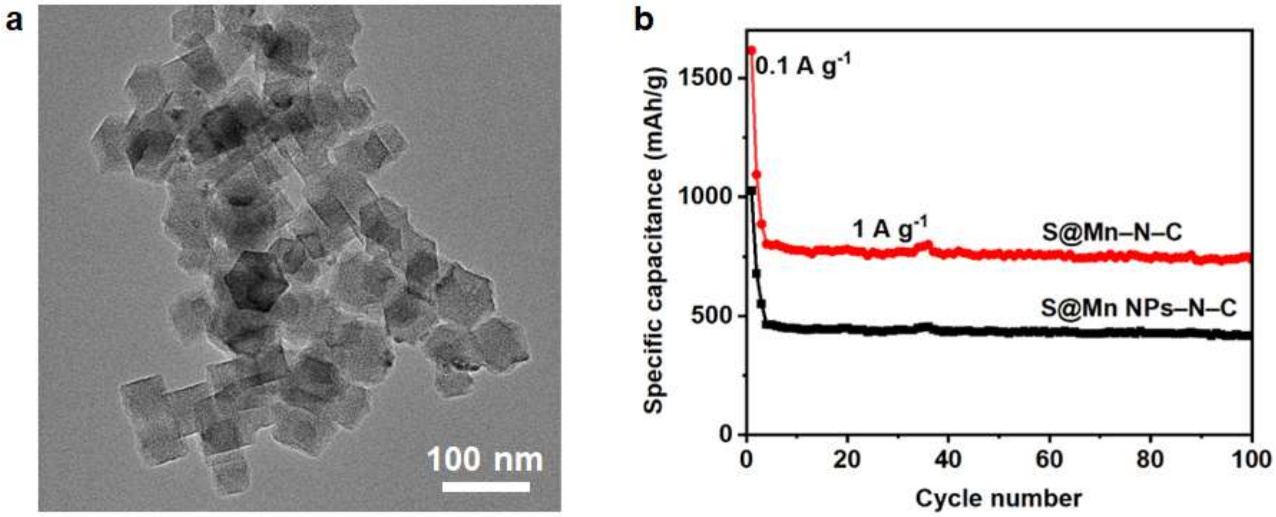

Figure S30. (a) TEM image of Mn NPs-N-C, (b) Cycling performance of S@Mn-N-C and S@Mn NPs-N-C electrode at 1 A/g for 100 cycles.

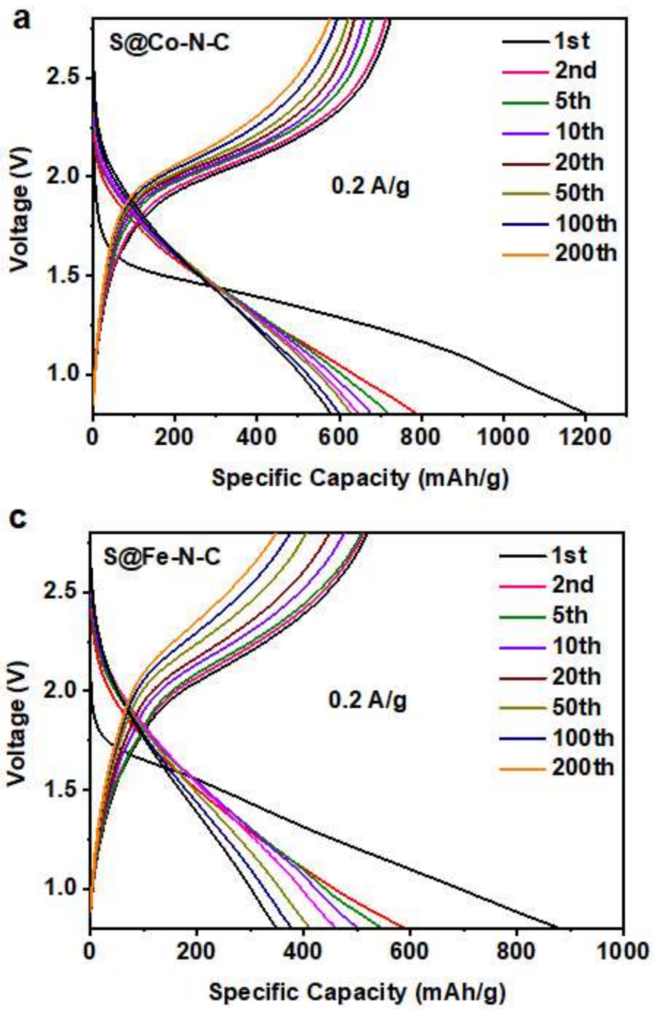

Figure S31. The discharge-charge voltage profiles of (a) S@Co-N-C, (b) S@Ni-N-C and (c) S@Fe-N-C electrodes at the current densities of 0.2 A/g.

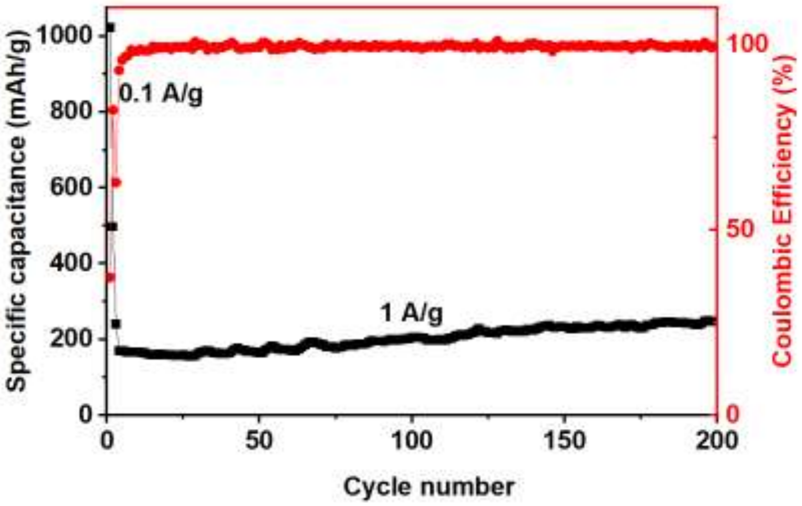

Figure S32. Cycling performance and Coulombic efficiency for S@Mn-N-C with areal loading of sulfur about 5 mg/cm^2 per CR2032 coin-cell at 1A/g for 200 cycles.

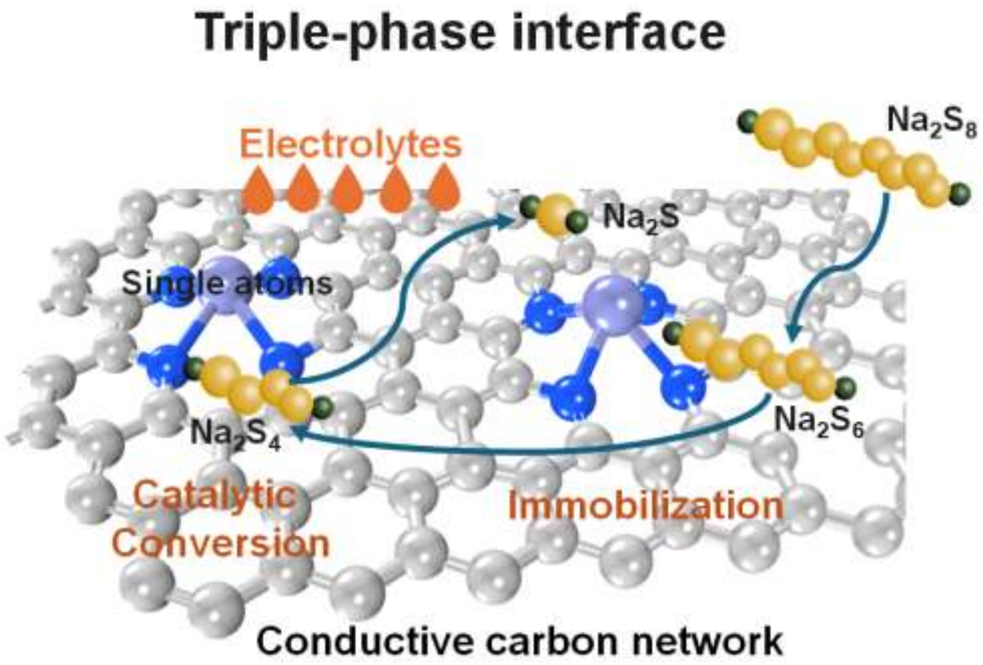

Figure S33. Schematic illustration of polysulfide redox reaction and Na_2S nucleation.

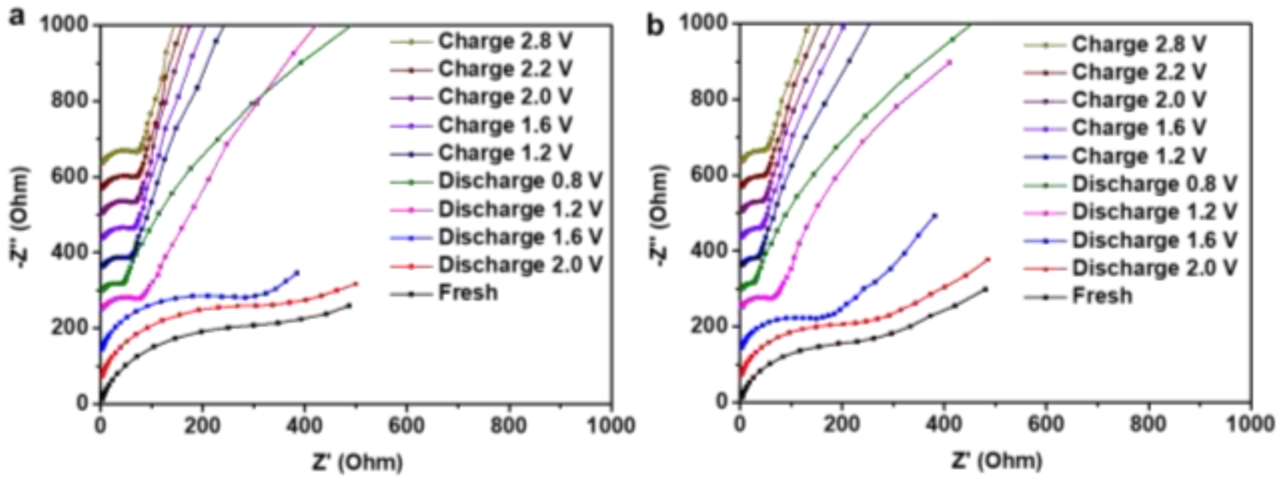

Figure S34. (a) EIS data of S@Mn-N-C electrodes at various discharging/charging stages. (b) EIS data of S@Cu-N-C electrodes at various discharging/charging stages.

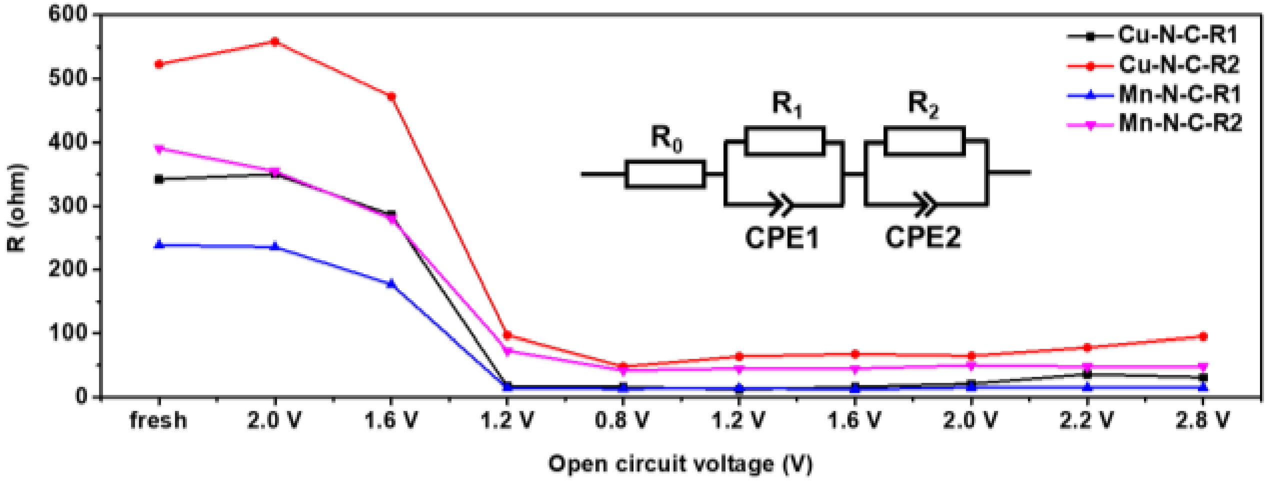

Figure S35. The Ohmic (R0), interfacial (R1), charge transfer (R2) resistances of S@Mn-N-C and S@Cu-N-C electrodes plotted versus various discharging/charging stages and inset is the equivalent circuit for fitting.

In the equivalent circuits, R_0 represents the impedance, which is mainly derived from the resistance of the electrolyte, R_1 is the resistance of the SEI film, and R_2 is the charge-transfer resistance at the conductive agent interface. CPE1 (Constant phase element) describes the space charge capacitance of the SEI, whereas CPE2 represents the double-layer capacitance (C_{dl}).

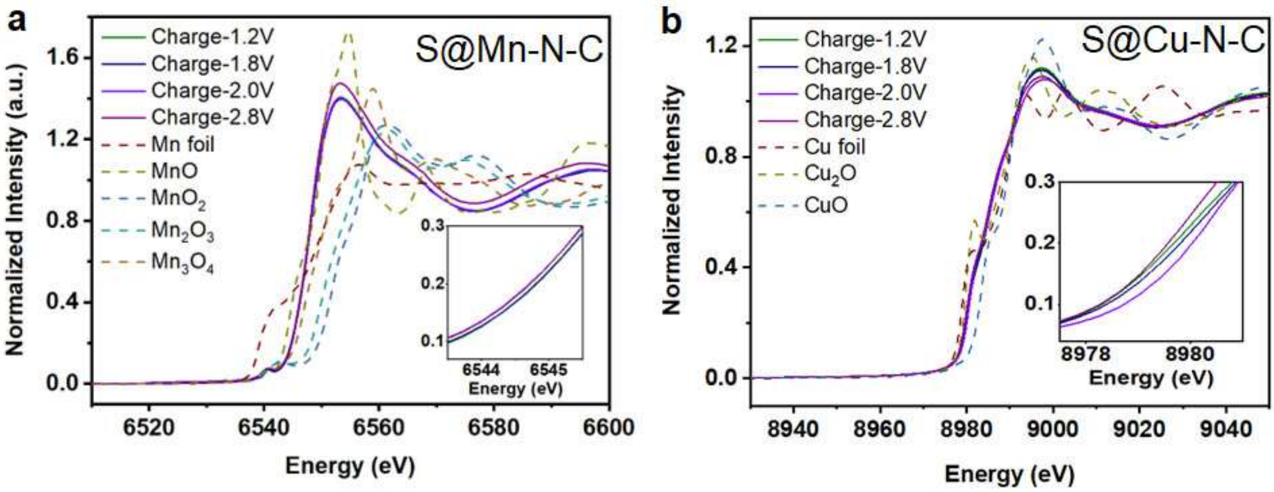

Figure S36. (a) The Mn K-edge from ex-situ XANES spectra of an S@Mn-N-C electrode during charging. (b) The Cu K-edge from ex-situ XANES spectra of an S@Cu-N-C electrode during charging. (The insets are details of the absorption energy shifts of white line peak).

Table S1. Physical properties of the prepared SACs.

SACs	S_{BET} [a] (m^2/g)	S_{micro} [b] (m^2/g)	S_{micro}/S_{meso} [b]	Pore size distribution [c] (nm)	V_t [d] (cm^3/g)	V_{micro} [e] (m^3/g)	V_{micro}/V_{meso} [f]	Metal content [g] (%)
Mn-N-C	845	622	2.8	1.07, 1.89, 3.81	0.57	0.49	6.1	0.88
Cu-N-C	805	488	1.6	0.6, 0.8, 1.2, 1.6, 3.5	0.70	0.26	1.7	0.93
Fe-N-C	832	486	1.4	0.48, 0.60, 0.81, 1.19,1.58, 2.75	0.71	0.26	1.7	0.84
Co-N-C	702	343	1.0	0.69, 0.85, 1.16, 1.51, 3.4	0.50	0.19	1.6	0.87
Ni-N-C	844	342	1.5	0.69, 1.18, 1.48, 2.7	0.72	0.19	2.8	0.85

[a] S_{BET} is the Brunauer-Emmett-Teller (BET) specific surface area.
[b] S_{micro} is the t-plot-specific micropore surface area calculated from the N_2 adsorption-desorption isotherm.
[b] S_{meso} is the specific mesopore surface area estimated by subtracting S_{micro} from S_{BET}.
[c] Pore size distribution is obtained by using DFT method.
[d] V_t is the total specific pore volume determined by using the adsorption branch of the N_2 isotherm at P/P_0=0.99.
[e] V_{micro} is the t-plot-specific micropore volume calculated from the N_2 adsorption-desorption isotherm.
[f] V_{meso} is the specific micropore volume calculated by subtracting V_{micro} from V_t.
[g] Metal content is determined by ICP-OES.

Table S2. Structural parameters of the prepared TM-based SACs (TM: Mn, Fe, Co, Ni and Cu) extracted from EXAFS fitting.

Samples	Scattering pair	CN	σ^2 (Å^2)	ΔE_0	R (Å)	R factor
Mn-N-C	Mn-N	4	0.003	-6.167	1.90	0.038
Fe-N-C	Fe-N	4	0.007	-7.776	1.99	0.0034
Co-N-C	Co-N	4	0.003	-8.722	1.89	0.015
Ni-N-C	Ni-N	4	0.005	-7.270	1.86	0.0041
Cu-N-C	Cu-N	4	0.007	-6.074	1.94	0.020

CN is the coordination number.

R is interatomic distance (the bond length between central atoms and surrounding coordination atoms).

σ^2: Debye-Waller factor (a measure of thermal and static disorder in absorber-scatterer distances).

ΔE_0 is edge-energy shift (the difference between the zero kinetic energy value of the sample and that of the theoretical model.

R factor is used to value the goodness of the fitting.

Table S3. Comparison of the cathode materials and electrochemical performances for room-temperature sodium–sulfur batteries.

Sample	Voltage range (V)	Reversible capacity (mAh/g)/Current	Rate capability (mAh/g)/ Current	Reference

		density (A/g)/Cycle number	density(A/g)	
S@Fe-HC	0.8-2.8	394/0.1/1000	820/0.1, 498/0.2, 383/0.5,313/1, 269/2, 220/5	8
S@Co_n-HC	0.8-2.8	292/0.1/200	391/0.1, 386/0.2, 352/0.5,305/1, 174/2, 127/5	9
CN/Au/S	0.8-2.8	369/10/2000	1010/0.1, 830/0.2, 755/0.5,678/1, 599/2, 532/5	10
MMPCS-800 @S	0.8-2.8	777/0.5/500 420/2/2000	1119/0.1, 977/0.2, 872/0.5,786/1, 688/2, 470/5	11
S/Ni-MOF-2D	0.5-2.8	347/3.2/1000	516/0.16, 416/0.32, 372/0.8,331/1.6, 284/3.2	12
NiS_2@NPCTs/ S	0.8-2.8	401/1/750	760/0.1, 691/0.2, 557/0.5,457/1, 346/2, 203/5	13
FeS_2@NCMS/ S	0.8-2.8	524/0.1/300	624/0.1, 573/0.2, 533/0.5,444/1, 340/2, 139/5	14
S/Mo_2N–W_2N@PC	0.8-3	799/0.2/100 517/1/400	915/0.2, 749/0.5, 619/1, 417/2, 190/5	15
FL-MoS_{2-x}@H C	0.8-2.8	514/0.1/100 341/1/1000	723/0.1, 554/0.2, 491/0.5,452/1, 416/2, 336/5	16
CFC/S@FC-P PY	1.0-2.5	700/0.2/200	1071/0.1, 962/0.2, 803/0.3,555/1, 441/2	17
SC-BDSA	0.6-2.8	590/0.25/200 452/2.5/1000	600/0.1, 500/0.4, 450/3.2	18
CFC/S-2	1.2-2.8	120/0.16/300	491/0.08, 265/0.16, 141/0.32,80/0.4, 48/1.6	19
S@Co_1-CoS_2/ NC	0.8-2.8	642/0.2/150	941/0.1, 756/0.2, 665/0.5,593/1, 533/2, 443/5	20
S@Mn-N-C	0.8-2.8	0.2/200/894 232/10/5000	1162/0.1, 937/0.2, 837/0.5,750/1, 650/2, 511/5, 348/10	**This work**

Table S4. Calculated ZPE and TS values of S, Na, and adsorbed species on the surfaces.

Species	ZPE (eV)	-TS (eV)
S (monomer S_8)	0.04	-0.08
Na (crystal)	0.02	-0.12
$^*S_8$	0.35	-0.72
$^*Na_2S_8$	0.37	-1.07
$^*Na_2S_6$	0.31	-0.85
$^*Na_2S_4$	0.20	-0.59
$^*Na_2S_2$	0.12	-0.47
$^*Na_2S$	0.07	-0.32
*NaS+*Na	0.07	-0.42

References:

1. G. Kresse, J. Hafner, Ab Initio Molecular-Dynamics Simulation of the Liquid-Metal-Amorphous-Semiconductor Transition in Germanium. Physical Review B, 1994, 49, 14251-14269.
2. G. Kresse, D. Joubert, From Ultrasoft Pseudopotentials to the Projector Augmented-Wave Method. Physical Review B, 1999, 59, 1758-1775.
3. G. Kresse, J. Furthmüller, Efficient Iterative Schemes for Ab Initio Total-Energy Calculations Using a Plane-Wave Basis Set. Physical Review B, 1996, 54, 11169-11186.
4. J. P. Perdew, K. Burke, M. Ernzerhof, Generalized Gradient Approximation Made Simple. Physical Review Letters, 1996, 77, 3865–3868.
5. S. Grimme, J. Antony, S. Ehrlich, H. Krieg, The Journal of Chemical Physics, 2010, 132, 154104.
6. H. J. Monkhorst, J. D. Pack, Special Points for Brillouin-Zone Integrations. Physical Review B, 1976, 13, 5188-5192.
7. G. Henkelman, B. P. Uberuaga, H. Jonsson, A Climbing Image Nudged Elastic Band Method for Finding Saddle Points and Minimum Energy Paths. The Journal of Chemical Physics, 2000, 113, 9901-9904.
8. B.-W. Zhang, T. Sheng, Y.-X. Wang, S. Chou, K. Davey, S.-X. Dou, S.-Z. Qiao, Angewandte Chemie International Edition, 2019, 58, 1484-1488.
9. Y.-X. Wang, J. Yang, W. Lai, S.-L. Chou, Q.-F. Gu, H. K. Liu, D. Zhao, S. X. Dou, Journal of the
   American Chemical Society, 2016, 138, 16576-16579.
10. N. Wang, Y. Wang, Z. Bai, Z. Fang, X. Zhang, Z. Xu, Y. Ding, X. Xu, Y. Du, S. Dou, G. Yu, Energy & Environmental Science, 2020, 13, 562-570.
11. C. Wu, Y. Lei, L. Simonelli, D. Tonti, A. Black, X. Lu, W.-H. Lai, X. Cai, Y.-X. Wang, Q. Gu, S.-L. Chou, H.-K. Liu, G. Wang, S.-X. Dou, Advanced Materials, 2022, 34, 2108363
12. C. Ye, Y. Jiao, D. Chao, T. Ling, J. Shan, B. Zhang, Q. Gu, K. Davey, H. Wang, S.-Z. Qiao, Advanced Materials, 2020, 32, 1907557.
13. Z. Yan, J. Xiao, W. Lai, L. Wang, F. Gebert, Y. Wang, Q. Gu, H. Liu, S.-L. Chou, H. Liu, S.-X. Dou, Nature Communications, 2019, 10, 4793.
14. Z. Yan, Y. Liang, J. Xiao, W. Lai, W. Wang, Q. Xia, Y. Wang, Q. Gu, H. Lu, S. L. Chou, Advanced Materials, 2020, 32, 1906700.
15. S. Zhang, Y. Yao, X. Jiao, M. Ma, H. Huang, X. Zhou, L. Wang, J. Bai, Y. Yu, Advanced Materials, 2021, 33, 2103846.
16. S. Luo, J. Ruan, Y. Wang, J. Hu, Y. Song, M. Chen, L. Wu, Small, 2021, 17, 2101879.

17. Z. Huang, B. Song, H. Zhang, F. Feng, W. Zhang, K. Lu, Q. Chen, Advanced Functional Materials, 2021, 31, 2100666.
18. T. Wu, M. Jing, L. Yang, G. Zou, H. Hou, Y. Zhang, Y. Zhang, X. Cao, X. Ji, Advanced Energy Materials, 2019, 9, 1803478.
19. Q. Lu, X. Wang, J. Cao, C. Chen, K. Chen, Z. Zhao, Z. Niu, J. Chen, Energy Storage Materials, 2017, 8, 77-84.
20. Y. Lei, C Wu, X. Lu, W. Hua, S. Li, Y. Liang, H. Liu, W. Lai, Q. Gu, X. Cai, N. Wang, Y. Wang, S. Chou, H. Liu, G. Wang, S.-X. Dou, Angewandte Chemie International Edition, 2022, 61, e202200384.
21. H. Zhang, M. Wang, X.-L. Huang, S. Lu, K. Lu, X. Wu, CCS Chemistry, 2024, 0, 1-16.
22. E. Zhang, X. Hu, L. Meng, M. Qiu, J. Chen, Y. Liu, G. Liu, Z. Zhuang, X. Zheng, L. Zheng, Y. Wang, W. Tang, Z. Lu, J. Zhang, Z. Wen, D. Wang, Y. D. Li, Journal of the American Chemical Society, 2022, 144, 18995−19007.
23. H. Zhang, B. Song, W. Zhang, B. An, L. Fu, S. Lu, Y. Cheng, Q. Chen, K. Lu, Angewandte Chemie International Edition, 2023, 62, e202217009.
24. D. Guo, J. Wang, T. Lai, G. Henkelman, A. Manthiram, Advanced Materials, 2023, 35, 2300841. 酥皮批
